# Functional genomics reveals the toxin–antitoxin repertoire and AbiE activity in *Serratia*

**DOI:** 10.1099/mgen.0.000458

**Published:** 2020-10-19

**Authors:** Hannah G. Hampton, Leah M. Smith, Shaun Ferguson, Sean Meaden, Simon A. Jackson, Peter C. Fineran

**Affiliations:** ^1^​ Department of Microbiology and Immunology, University of Otago, Dunedin 9054, New Zealand; ^2^​ Genetics Otago, University of Otago, Dunedin 9054, New Zealand; ^3^​ Bio-protection Research Centre, University of Otago, Dunedin 9054, New Zealand

**Keywords:** AbiE, abortive infection, toxin–antitoxin, transposon mutagenesis

## Abstract

Bacteriophage defences are divided into innate and adaptive systems. *Serratia* sp. ATCC 39006 has three CRISPR-Cas adaptive immune systems, but its innate immune repertoire is unknown. Here, we re-sequenced and annotated the *Serratia* genome and predicted its toxin–antitoxin (TA) systems. TA systems can provide innate phage defence through abortive infection by causing infected cells to ‘shut down’, limiting phage propagation. To assess TA system function on a genome-wide scale, we utilized transposon insertion and RNA sequencing. Of the 32 TA systems predicted bioinformatically, 4 resembled pseudogenes and 11 were demonstrated to be functional based on transposon mutagenesis. Three functional systems belonged to the poorly characterized but widespread, AbiE, abortive infection/TA family. AbiE is a type IV TA system with a predicted nucleotidyltransferase toxin. To investigate the mode of action of this toxin, we measured the transcriptional response to AbiEii expression. We observed dysregulated levels of tRNAs and propose that the toxin targets tRNAs resulting in bacteriostasis. A recent report on a related toxin shows this occurs through addition of nucleotides to tRNA(s). This study has demonstrated the utility of functional genomics for probing TA function in a high-throughput manner, defined the TA repertoire in *Serratia* and shown the consequences of AbiE induction.

## Data Summary

Sequencing data is available in the National Center for Biotechnology Information (NCBI) SRA database under accession number PRJNA637196. The assembled genomes are available under NCBI RefSeq accession numbers NZ_CP025084.1 (* Serratia* sp. ATCC 39006 WT) and NZ_CP025085.1 (* Serratia* sp. ATCC 39006 LacA – *lacZ-* derivative). Supplementary figures and tables are included with the online version of this article.

**Impact Statement**

There has been a surge of research into bacteriophage defences in bacteria, which are broadly classified into innate and adaptive systems. One important class of innate defence systems are the toxin–antitoxin (TA) modules, which have diverse functions including conferring phage resistance via abortive infection. To gain a holistic view of how defence mechanisms protect a particular bacterium, the collection of defence systems within the strain must be identified. Here, we combined bioinformatics and high-throughput transposon insertion sequencing to identify and determine the activity of TA systems in *Serratia* sp. ATCC 39006 on a genome-wide scale. One poorly characterized but widespread TA family (AbiE) was then characterized further. Our analyses demonstrate that the nucleotidyltransferase AbiE toxin in *Serratia* affects cellular tRNA abundance and inhibits cell growth. We propose that *Serratia* AbiE functions similarly to a homologous system in *Mycobacterium tuberculosis *, which was shown recently to inhibit translation and growth by transferring nucleotides to tRNAs. Our study shows that by combining genomic and functional genomic techniques, functional TA systems can be predicted in a high-throughput manner.

## Introduction

Bacteria are constantly faced with viral predation and have evolved multiple mechanisms to protect themselves [1]. For example, CRISPR-Cas adaptive immunity provides a sequence-specific memory of past invaders through recognition of foreign nucleic acids [2]. Another mechanism is abortive infection (Abi), which provides population-level immunity [1, 3]. During abortive infection, phages adsorb but infection is followed by bacterial dormancy or death; hence, phage propagation is inhibited and few, if any, phages are released [1, 3]. The phenotypic and descriptive definition of abortive infection has resulted in a diverse set of known Abi systems that have a wide range of molecular mechanisms [3, 4]. Some Abi systems function via a two-gene toxin–antitoxin (TA) mechanism, such as ToxIN/AbiQ [5, 6] and AbiE [7, 8]. Some TA systems in *Escherichia coli* can also elicit phage resistance, most likely via abortive infection [9–12].

TA systems were first discovered due to their ability to stabilize plasmids, since bacterial cells that lose the plasmid following cell division experience either growth cessation or death [13]. Subsequently, TA loci were shown to have diverse roles, including assisting bacteria in adaptation to stress, phage resistance and providing superinfection exclusion when encoded in prophage genomes [14, 15]. TA systems are commonly composed of a stable toxin, which inhibits cellular growth, and a labile antitoxin, which counteracts toxicity [16]. Under conditions that lead to degradation of the antitoxin such as cell stress, or when nascent antitoxin synthesis ceases, the stable toxin elicits deleterious effects. There are seven different types of TA system (I–VII) based on the nature of the antitoxin and toxin, and the interaction, or lack thereof, between the components [14, 17]. However, it has been suggested that the current classification scheme is not reflective of TA diversity, as the same system may be assigned to more than one type [18].

There is a growing interest in how multiple phage defence systems function together in a single bacterium [19]. For example, restriction-modification systems and CRISPR-Cas act synergistically to resist phages [20]. Additionally, TA systems are proposed to function alongside CRISPR-Cas. In this scenario, TA systems might function either as a kill switch if CRISPR-Cas fails, or as a cell-slowing mechanism to assist in adaptation to, and clearance of, the invader [21].

*Serratia* sp. ATCC 39006 (herein referred to *Serratia *) is a Gram-negative bacterium that has been used as a model to study phage–host interactions and the role of its three functional CRISPR-Cas systems – types I-E, I-F and III-A [22–24]. In contrast, we currently know little about the innate immune repertoire of *Serratia *.

Here, we examine the TA arsenal of *Serratia* by applying several sequencing-based approaches: genome sequencing, transposon insertion sequencing (TIS) and RNA sequencing (RNA-seq). We demonstrate the presence of 32 TA loci, including types I [25], II [13], IV [7], V [26] and VII [27]. Through our high-density transposon mutagenesis and gene essentiality screen, 11 TA loci were confirmed to be toxic in the absence of the antitoxin, including three type IV systems of the AbiE family. Using RNA-seq, we uncovered evidence that this toxin family – that functions through a nucleotidyltransferase mechanism – causes tRNA dysregulation, likely resulting in decreased translation in the cell and bacteriostasis. Our results are consistent with the mechanism of an AbiE toxin homologue that was recently shown to add pyrimidines to the 3′-CCA acceptor stem of specific tRNAs [28] and suggest this mode of toxicity is conserved in AbiE proteins.

## Methods

### Strains, plasmids and growth conditions

*Serratia* sp. ATCC 39006 strain LacA was used, unless stated otherwise, and was grown at 30 °C in lysogeny broth (LB) with shaking at 200 r.p.m. or on LB containing 1.5 % (w/v) agar (LBA). *E. coli* DH5α was used for cloning and *E. coli* ST18 for conjugative transfer of plasmids to *Serratia. E. coli* strains were grown at 37 °C in LB, with 5-aminolevulinic acid (ALA; 50 µg ml^−1^) for ST18 derivatives. When relevant, media were supplemented with the following antibiotics and supplements: 25 µg chloramphenicol ml^−1^, 100 µg ampicillin (Ap) ml^−1^, 50 µg kanamycin (Km) ml^−1^, 0.1 or 0.5 % (w/v) l-arabinose (Ara) (as indicated), 0.2 % (w/v) d-glucose and 1 mM IPTG. Bacterial cell turbidity was measured in a Jenway 6300 spectrophotometer at 600 nm (OD_600_). DNA from PCR was purified using the Illustra GFX PCR DNA and gel band purification kit (GE Healthcare). Plasmid DNA was isolated using the Zyppy plasmid miniprep kit (Zymo Research). The strains, plasmids and oligonucleotides used in this study are listed in Tables S1–S3 (available with the online version of this article), respectively.

### Plasmid construction

Standard molecular biology techniques were used unless otherwise stated. Primers PF1601+PF1602, PF1603+PF1604 and PF1605+PF1606 were used to amplify AbiE-A2, -A1, and -A3 regions, respectively, from LacA genomic DNA. Fragments were digested and cloned into pPF260. For toxin plasmids, primers PF1607+PF1608, PF1609+PF1610 and PF1611+PF1612 were used to amplify toxin regions for AbiE-T2, -T1 and -T3, respectively. For constructs with the native ribosome binding site (RBS), primers PF1679+PF1610 and PF1680+PF1612 were used to amplify AbiE-T1 and -T3, respectively. For constructs with both the antitoxin and toxin, PF1603+PF1610 and PF1605+PF1612 were used to amplify AbiE-1 and AbiE-3, respectively. Digested fragments were cloned into pBAD30 and these plasmids were used to transform *E. coli* in the presence of glucose repression to prevent toxicity. The cloned inserts of plasmids constructed in this study were confirmed by DNA sequencing, performed by Massey Genome Service, Massey University, New Zealand, and analysed using Geneious (version 2019.2.1) (https://www.geneious.com).

### Genome sequencing

Genomic DNA was extracted from *Serratia* sp. ATCC 39006 (WT) [29] and *Serratia* LacA (a *lacZ* derivative of *Serratia* WT generated by ethyl methanesulfonate mutagenesis (EMS) [30]) using a cetyltrimethylammonium bromide (CTAB) and organic extraction protocol. Briefly, cells were washed twice with 5M NaCl then lysed using lysis buffer (0.05 mM Tris, 0.4 mM NaCl, 0.02 mM EDTA, 2 % SDS and 1 mg proteinase K) and CTAB buffer (10 mM Tris, 2 mM EDTA, 0.14 M NaCl, 2 g CTAB per 100 ml) at 65 °C for 30 min. The lysate was extracted twice with chloroform : isoamyl alcohol, then the nucleic acids were precipitated with ethanol and resuspended in TE buffer. RNA was removed by treatment with RNase (0.02 mg) for 30 min at 37 °C, which was removed by chloroform : isoamyl alcohol extraction and precipitation of the genomic DNA (gDNA) with sodium acetate and ice-cold ethanol. The pellet was washed twice with ethanol, then resuspended in 250 µl TE buffer. WT genomic DNA was sequenced by PacBio RS II (Macrogen), and both the WT and LacA strains by Illumina HiSeq (Macrogen). The coverage of the WT by Illumina sequencing was 330-fold and of the LacA strain was 465-fold. The sequence was assembled and polished using Canu v1.3 and Pilon v1.21.

### Genome annotation

The assembled genomes were uploaded to the National Center for Biotechnology Information Prokaryotic Genome Annotation Pipeline (PGAP) for annotation and are available under RefSeq accession numbers NZ_CP025084.1 (WT *Serratia *) and NZ_CP025085.1 (LacA *Serratia *). Prodigal [31] was used for confirmation of coding region boundaries. The *lacZ* derivative (LacA) of *Serratia* was previously derived by EMS [30], and 10 single nucleotide mutations between the *Serratia* WT and LacA genomes were identified (Table S4). Phaster [32] and IslandViewer [33] were used to predict prophages and genomic islands. TA systems within *Serratia* were identified computationally, using the PGAP annotations in combination with RASTA-Bacteria (rapid and automated scan for toxins and antitoxins) [34], TASmania [35] and PADS arsenal [36]. Clustal Omega was used for protein alignments [37].

### Transposon mutagenesis

*Serratia* PCF396 (* Serratia* LacA with the pigment operon deleted so the strain is compatible with fluorescent reporters) was mutagenized with a mini-Tn*5* derivative by conjugation from *E. coli* ST18 carrying pKRCPN2 as follows. Five millilitre overnight cultures of each strain were standardized to an OD_600_ of 1. Equal ratios of these were mixed and 100 µl of the mixed culture was spotted on 0.22 µm pore nitrocellulose filters on LBA+ALA. Plates were incubated for 6 h at 30 °C for mutagenesis to occur. Following this, the cells were recovered by vortexing the filters in 15 ml LB. The resuspended cells were then used to inoculate 2 l flasks containing 500 ml LB to a starting OD_600_ of 0.02. After 24 h incubation at 30 °C with shaking, a sample was taken for gDNA extraction to identify transposon mutants that may have a significant growth defect. This gDNA was extracted using the DNeasy blood and tissue kit (Qiagen), following the manufacturer’s instructions. The remaining cells were harvested by centrifugation at 3000 ***g*** for 15 min. Harvested cells were resuspended in LB to an OD_600_ of 3, then diluted 50 % in glycerol to give a final OD_600_ of 1.5 in 1 ml and frozen at −80 °C. To reduce *E. coli* present*,* a second outgrowth step was performed, where the 1 ml sample stock was subcultured in 29 ml LB in a baffled flask with Km (to select for the transposon). Samples were then incubated for 16 h at 30 °C with shaking. A 1 ml sample was then used for genomic DNA extraction as per the earlier sample. The DNA concentrations of all samples were quantified using the Qubit for HS DNA (Thermo Fisher Scientific).

### Library preparation and sequencing

The NEB Next Ultra II FS kit for Illumina was used for library preparation following the protocol for inputs <100 ng. Modifications from the manufacturer’s instructions were as follows. Two rounds of PCR for enrichment were implemented, round one used custom PCR primers PF3140 and PF3139 (a biotinylated primer). Biotinylated products were captured using Dynabeads M-270 Streptavidin (Invitrogen) following the manufacturer’s instructions, and the bead-bound DNA served as the template in the second round PCR, with primer PF3270 and an indexing primer (NEBNext multiplex oligos for Illumina). Library quality and concentration were assessed using a Qubit HS DNA assay (Thermo Fisher Scientific), a Bioanalyzer high sensitivity DNA assay (Agilent) and quantitative PCR (qPCR, KAPA library quantification kit). Primers PF3124 and PF3125 were used to determine the molarity of library fragments containing Illumina flow cell binding sites, while primers PF2926 and PF3125 were used to determine the molarity of fragments containing transposon junctions. Samples that passed the above quality control parameters were sent in 10 nM aliquots to the Otago Genomics Facility, University of Otago, New Zealand. Here, samples were pooled with 10 % PhiX (control library) and loaded at 1.5 pM (for low diversity) using a MiSeq reagent kit v3 150 cycle kit for Illumina. Libraries were subject to single-end sequencing for 75 cycles using primers PF2926 (to sequence transposon junctions) and PF3441 (Illumina Read 1 primer – to sequence the PhiX control). The standard Illumina primer mix was used for index sequencing. Sequencing with PF2926 generates a 12 nt transposon ‘tag’ to verify reads originate from transposon junctions.

### Data analysis for TIS mapping

Demultiplexed reads were provided by the Otago Genomics Facility. Trimmomatic [38] was used for the trimming of Illumina adaptors. Trimmed reads were mapped to the LacA genome using the Bio-Tradis pipeline (https://github.com/sanger-pathogens/Bio-Tradis) [39]. TraDIS filters reads containing the transposon tag and then maps these transposon-junction reads to the chromosome. The result is a summary file for mapping and insertion statistics. In addition, insert site ‘plot’ files are generated for each sample, which indicate the number of reads at each nucleotide position. The bacteria_tradis script was run with the parameters ‘--smalt --smalt_k 10 --smalt_s 1 --smalt_y 0.92 --smalt_r 1 mm 2 v -f filelist.txt -t TATAAGAGACAG -r LacA.fasta’ (--smalt defines mapping parameters and -t defines transposon tag). To convert the output file into tabular format, tradis_gene_insert_size was used with ‘-trim3 0.1 laca.embl’, which assigns the insertions to features listed in the embl file, while trim3 0.1 discards insertions falling in the 10 % from the 3′ end. The output file was then analysed in the R environment [40] to determine genes that are essential, ambiguous and non-essential. The essentiality script was derived from published research [39], which functions to predict the essentiality of a gene through fitting insertion indices (the number of insertions divided by feature length) to a bimodal distribution [39, 41, 42]. Genes that cannot be assigned to essential or non-essential are deemed ambiguous.

### Sample collection for RNA-seq and RNA extraction

Overnight *Serratia* LacA cultures, containing either pBAD30 or pPF703 in triplicate, were used to inoculate 250 ml flasks containing 25 ml LB+Ap+glucose to a starting OD_600_ of 0.05. After 5 h growth at 30 °C with shaking at 200 r.p.m., bacteria were harvested via centrifugation, and washed two times in PBS to remove residual glucose. Bacteria were then resuspended in LB with 0.1 % Ara to induce the toxin and incubated at 30 °C with shaking at 200 r.p.m. At 5 and 20 min post-toxin induction with Ara, 1 ml samples were taken to measure the number of c.f.u. and 2 ml samples were taken for RNA extraction. For c.f.u. measurement, cells were harvested by centrifugation and then washed twice in PBS to remove excess Ara. A dilution series was performed in PBS and then plated onto LBA+Ap+glucose. After overnight incubation at 30 °C, c.f.u. numbers were determined for each treatment condition. For RNA-seq, cells were isolated by centrifugation and then resuspended in RNA*later* (Thermo Fisher Scientific). Samples were stored at −20 °C until required. Samples were removed from the RNA*later* by centrifugation. Total RNA was then isolated from cells using a Qiagen RNeasy kit. To ensure full cell lysis, an additional bead beating step of 30 s was included while the cells were in RLT (Qiagen RNeasy kit lysis buffer) and β-mercaptoethanol. Residual gDNA was removed using TurboDNase (Thermo Fisher Scientific) following the manufacturer’s protocol, except that after 30 min incubation with TurboDNase, another 2 µl DNase was spiked in and left to incubate for a further 30 min at 37 °C. The inactivation reagent was then used as per the protocol. RNA samples were confirmed to be gDNA-free by PCR with primers PF796 and PF797 to amplify the *flhDC* operon. Quality control checks were performed by assessing RNA on the NanoDrop One (Thermo Fisher Scientific) and 2100 Bioanalyzer (Agilent Genomics).

### RNA-seq and analysis

RNA samples were sequenced at Vertis Biotechnologie, where cDNA libraries were made for Illumina sequencing. Single-end 75 bp sequencing was performed using an Illumina NextSeq 500, with approximately 10 million reads obtained per library. The fastq files were run through Trimmomatic to remove adaptors and low-quality reads [38]. fastqc (Babraham Bioinformatics; http://www.bioinformatics.bbsrc.ac.uk/pro- jects/fastqc/) was run to evaluate the quality of the RNA-seq. Bowtie2 [43] with default parameters was used to align raw sequence reads to the LacA genome. The alignment was converted to BAM format using SAMtools [44]. DEseq2 [45], run in the R environment [40], was used to identify differentially expressed genes using a Wald test, followed by a Benjamini and Hochberg procedure. All sets were defined by a false discovery rate of 10 %. Visualization of sequencing reads and calculations of reads per kilobase million (RPKM) were performed in Artemis [46].

### Kill-rescue assays

For antitoxicity assays, the AbiE-A expression constructs, under control of the T5/*lac* promoter, were tested for their ability to abrogate AbiE-T-mediated toxicity, under the control of an Ara-inducible promoter [47]. *Serratia* cultures were grown overnight in a Labcon deep 96-square-well plate at 12 000 r.p.m. at 37 °C using an orbital plate shaker (IncuMix; BioProducts). These were subcultured into a fresh deep well plate containing 1.2 ml LB+Ap+ Km+glucose per well at a starting OD_600_ of 0.05. After 3 h of growth at 37 °C with shaking at 12000 r.p.m., bacteria were isolated by centrifugation, washed twice and a dilution series was performed in PBS. Toxicity was quantitated by plating the dilution series onto LBA+Ap+Km plates supplemented with: (i) glucose only; (ii) Ara only; and (iii) Ara and IPTG. After ~16 h incubation at 37 °C, the number of c.f.u. ml^−1^ was determined for each treatment.

### Toxin suppression by overexpression – ASKA (A complete Set of *Escherichia coli* K-12 ORF Archive) library

Due to frequent mutation of the *Serratia* AbiE-T2 expression plasmid in *E. coli *, the *Streptococcus agalactiae* AbiEii homologue was used for this experiment. Briefly, *E. coli* cells containing an Ara-inducible AbiEii*_Sag_* (pRLD12) were made competent via the Inuoue method [48]. The ASKA library, minus all Ara efflux genes except *ydeA,* was pooled into 16 sub-libraries and plasmids isolated from each pool. Each pool was used to transform via heat-shock competent *E. coli* containing AbiEii*_Sag_*. A positive control of pCA24N*ydeA* and a negative control of pCA24N were used in transformations at the same time. Transformation*s* were plated onto LBA+Ap+Ara (0.5 %, w/v)+chloramphenicol+IPTG to select for cells containing both the toxin and the ASKA library plasmids, in addition to inducing expression from both. Colonies that grew following the transformation were isolated, and PCR was used to amplify the unknown ORFs. PCR products were analysed by gel electrophoresis and those of interest were sent for sequencing. Basic local alignment search tool (blast) was used to identify genes within sequenced PCR products. To check for false positives, identified ‘hits’ were used to re-transform cells containing the toxin plasmid. No hits were able to protect from toxin expression upon re-transformation.

## Results

### *Serratia* contains 32 predicted TA systems

**Fig. 1.**
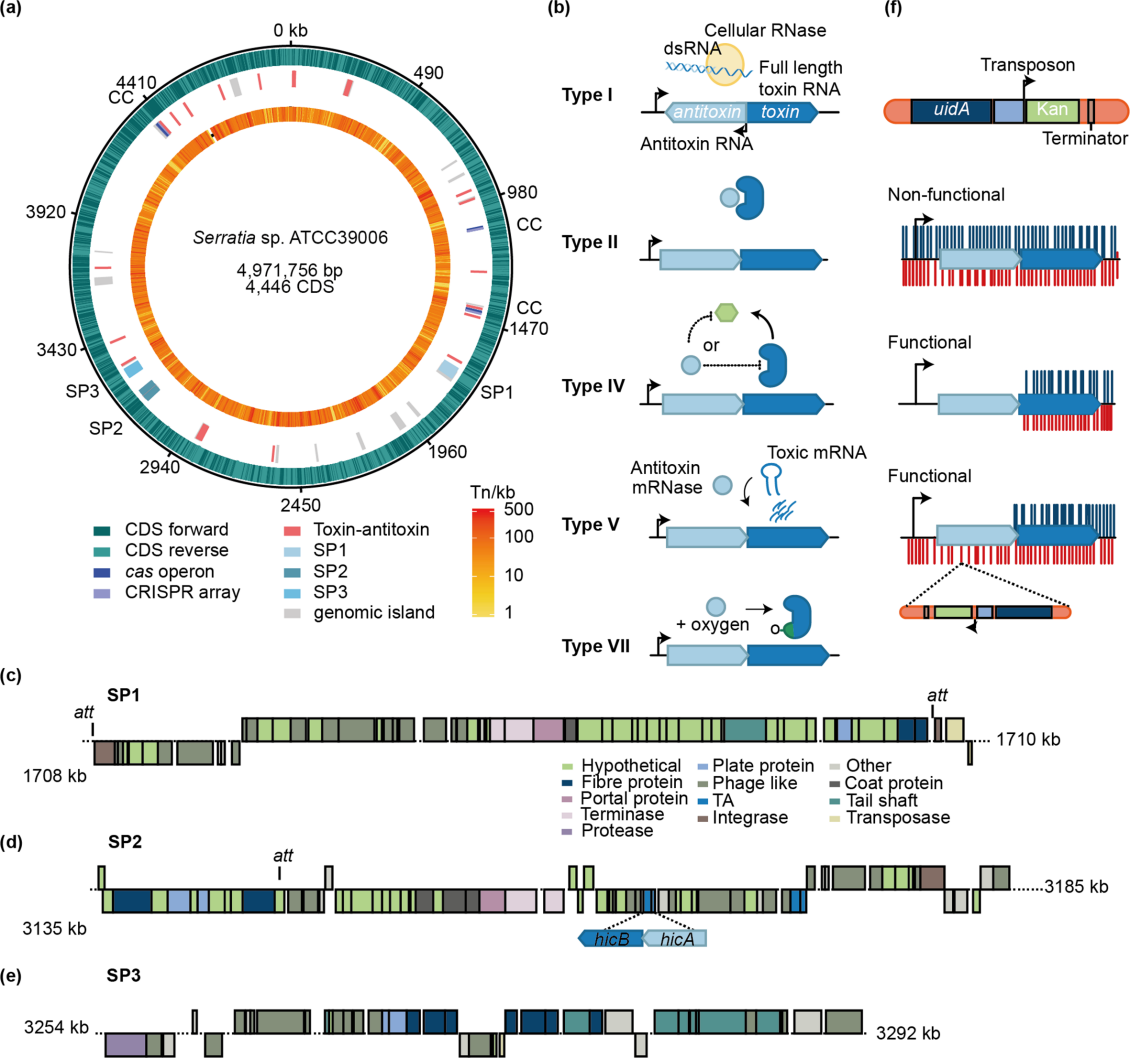
*
Serratia* contains 32 TA systems. (a) Genome map of *Serratia* showing locations of TA systems, *cas* operons and CRISPR arrays (CC), prophages and predicted genomic islands. The orange ring shows the 374 000 unique transposon insertions (scale: insertion density kb^−1^) CDS= coding sequence. (b) Models for type I, II, IV and V TA systems. (c, d and e) Schematics of prophages (c) SP1, (d) SP2 and (e) SP3 showing TA system locations. (f) The Tn*5* transposon used, and transposon insertion in a non-functional, a functional and a functional TA system with insertions in the reverse strand of the antitoxin that disrupt transcription across the operon and, thus, no toxin is transcribed.

To examine the TA repertoire in *Serratia* LacA, we combined genomic and functional genomic approaches. First, we generated a high-quality genome sequence and performed bioinformatic characterization of TA systems. The previous draft *Serratia* sp. ATCC 39006 sequence was incomplete [49]; therefore, we re-sequenced *Serratia* WT using long reads (PacBio) and error-corrected with short-read data (Illumina). The *Serratia* LacA (*lacZ* derivative) was also sequenced using Illumina and completed using the WT PacBio as the reference. This resulted in complete genomes of 4 971 757 kb with 4313 protein-encoding genes, 22 rRNAs (eight 5S, seven 16S and seven 23S), 74 tRNAs and 7 conserved non-coding RNAs (Fig. 1a). Ten single nucleotide changes were identified between *Serratia* WT and LacA (Table S4). By protein homology and gene synteny, we identified 32 putative TA systems (or individual TA components), comprising 7 type I (four of which resemble pseudogenes), 19 type II, 4 type IV, 1 type V and 1 type VII system (Fig. 1a, b, Table 1). This level of TA abundance is similar to both *E. coli* K-12 and *Salmonella enterica* Typhimurium [50, 51]. TA systems stabilize not only plasmids, but also other mobile elements, such as genomic islands and prophages [52]. Twenty-two genomic islands were predicted in *Serratia *, and 14 TA systems were located within five of these (Table S5). *Serratia* also has three predicted prophages that we named SP1 (for *Serratia* prophage 1), SP2 and SP3, which contained none, one and none TA systems, respectively (Fig. 1c–e, Table S6). Overall, roughly half of the diverse TA repertoire of *Serratia* is located in genomic islands or prophages.

**Table 1.** TA systems in *Serratia* sp. ATCC 39006

System	Locus tag (CWC46_no.)	**Fa**mily	Functional*	AT insertion index†	T insertion index†	AT RPKM‡	T RPKM‡	Notes
**Type I**								
*symE*-1	12 890–12 895	SymE/XRE-HTH	–	0	0	64.5	48.3	GI13 – duplicated region
*symE*-2	12 920–12 925	SymE/XRE-HTH	–	0	0	66.9	46.1	GI13 – duplicated region
*symE*-3	12 955	SymE pseudogene	–	na	1.85×10^−1^	na	Not expressed	Solo toxin, GI13
*symE*-4	12 985	SymE pseudogene	–	na	2.72×10^−1^	na	Not expressed	Solo toxin, GI13
*symE*-5	12 990	SymE pseudogene	–	na	3.91×10^−2^	na	Not expressed	Solo toxin, GI13
*symE*-6	13 010	SymE pseudogene	–	na	3.04×10^−2^	na	Not expressed	Solo toxin, GI13
*symE*-7	17 015	SymE	–	na	3.41×10^−1^	na	49	–
**Type II**								
*hipAB-1*§	00 045–00 050	HipAB	Functional	7.2×10^−3^	7.90×10^−2^	26.4	51.9	–
*hipAB-2*	01 115–01 110	HipAB	Functional	3.5×10^−3^	8.08×10^−2^	25.2	36.3	GI1
*hipAB-3*	01 145–00 150	HipAB	Functional	3.8×10^−3^	6.00×10^−2^	21.2	15.9	GI1
*hipAB-4*	07 540–07 545	HipAB	Functional	0	5.10×10^−2^	107.1	56.4	–
*hipAB-5*	20 605–20 610	HipAB	–	4.0×10^−2^	1.37×10^−1^	22.8	30.7	–
*relBE/parDE-1*	00 060–00 065	RelEB/ParED	–	4.7×10^−2^	2.4×10^−2^	215	216	–
*relBE/parDE-2*	00 090–00 095	RelEB/ParED	–	7.1×10^−2^	3.6×10^−2^	76.4	80.9	–
*relBE/parDE-3*	04 410–04 415	RelEB/ParED	Functional	0	3.4×10^−2^	244.6	199.6	GI4
*relBE/parDE-4*§	19 965–19 970	RelEB/ParED	Functional	3.5×10^−2^	3.5×10^−2^	123.7	137.8	GI18
*relBE/parDE-5*	21 320–21 325	RelEB/ParED	–	5.6×10^−2^	3.6×10^−2^	97.45	80.39	–
*vapBC-1*§	01 090–01 095	VapC, IrrE-like HTH domain	Functional	1.6×10^−2^	9.5×10^−2^	40.5	60	GI1
*vapC-2*	22 100	VapC	–	na	1.5×10^−1^	na	157	Only T
*phd-1*	05865	Phd	–	0	na	3507	na	Only AT
*hicAB-1*	06 505–06 510	HicAB	–	3.6×10^−2^	1.8×10^−2^	503.1	488.5	–
*hicAB-2*	14 305–14 310	HicAB	Functional	2.7×10^−3^	3.1×10^−2^	1090	6125.7	SP2
*parDE-1*	13 000–13 005	ParED	–	6.8×10^−2^	9.9×10^−2^	18.1	25.9	–
*higAB-1*	15 105–15 110	HigBA	–	2.3×10^−2^	1.1×10^−1^	116.6	62.4	GI15
*higAB-2*	15 590–15 595	HigBA	–	4.3×10^−2^	2.9×10^−2^	17.8	21.2	–
*ccdAB-1*	20 295–20300	CcdBA	–	4.5×10^−2^	5.8×10^−2^	89.9	101.7	–
**Type IV**								
*abiE-1*	01 120–01 125	AbiE	Functional	0	6.5×10^−2^	52.4	17.5	GI1
*abiE-2*	04 260–04 265	AbiE	Functional	1.4×10^−3^	1.5×10^−1^	15.3	21.3	–
*abiE-3*§	06 665–06 670	AbiE	Functional	2.9×10^−2^	5.7×10^−2^	18.7	8.86	–
*cbtA-yeeU-1*	19 815–19 820	CbtA-YeeU	–	5.6×10^−2^	5.4×10^−2^	0.96	1.66	GI18
**Type V**								
*ghoT-1*	11 680	GhoT	–	na	9.0×10^−2^	–	176.3	Only T
**Type VII**								
*hha-tomB-1*	18 870–18 875	Hha-TomB	–	1.8×10^−1^	2.2×10^−2^	513.7	256.6	–

*Predicted as being functional due to the criteria outlined in Fig. 1 and under the growth conditions tested.

†The ratio of transposon insertions to gene length, with low values indicating that few transposon insertions are tolerated. na signifies that despite the presence of a toxin or antitoxin, no partner component could be detected.

‡RPKM – relative expression from RNA-seq data. Not expressed refers to no detectable expression under the conditions tested.

§Genes identified as functional based upon strand bias by transposon insertion.

GI, Genomic island.

### TA validation by high-throughput functional genomics

Prediction of TA systems via sequence homology is not directly indicative of function. Therefore, we applied two complementary high-throughput methods to interrogate TA function under the same conditions: saturation transposon mutagenesis and high-throughput sequencing, termed TIS [53] and RNA-seq. Recent work has demonstrated that although stress induces transcription of a TA loci, it does not result in active toxin [54]. Antitoxin degradation or loss of expression is important for toxin activity. Therefore, we chose to mimic the loss of antitoxins by transposon (Tn) disruption under normal growth conditions in our analysis to identify active toxins. For TIS, we generated a library of ~374 000 mutants using a Tn*5* derivative and deep-sequenced the transposon insertion junctions (Fig. 1a). The transposon has leaky transcription through the transcriptional terminator downstream of the Km-resistance gene, which allowed us to exploit the transposon insertion orientation to uncover essential genes within operons (Fig. 1f) [55]. Transposon insertions in antitoxins of functional TA systems typically result in growth arrest and are depleted in the observed TIS dataset (Fig. 1c). Occasionally, insertions are observed only on the reverse strand of an antitoxin. This is indicative that any leaky transcription from, or through, the transposon inserted in the forward strand would result in transcription of the toxin, and growth arrest. In contrast, when inserted in the reverse orientation, convergent transcription occurring from the Tn promoter and the TA promoter prevents transcription and toxin production [56]. Transposon insertions within the antitoxins of non-functional TAs do not affect cell viability and result in similar insertion frequencies in the antitoxin and toxin (Fig. 1c). Overall, the TIS functional genomics approach indicated that at least 11 of the *Serratia* TA systems are functional under the growth conditions tested. In the following sections, we present key findings for the type I and various functional type II and IV TA systems.

### Dynamic evolution of SymE type I TA loci includes gene duplication

In *Serratia *, we identified seven type I toxins of the SymE family (Table 1). In *E. coli *, SymE evolved from AbrB/MazE-fold antitoxins and gained endonuclease activity [57]. The antitoxin, *symR*, is encoded antisense to the *symE* gene, resulting in a small RNA that inhibits toxin translation and promotes mRNA turnover by binding to the *symE* RBS [57]. In *Serratia *, *symE-7* encodes a full length SymE homologue that is expressed and tolerated transposon insertions (Fig. S1). Due to the overlapping antisense transcription of *symER* loci, any insertions in the unidentified antitoxins would also disrupt the production of functional toxin; therefore, their essentiality could not be determined.

The remaining six *Serratia symE* genes are within predicted genomic island 13 (Table S5). Furthermore, *symE-1* and *symE-2* are located within a genomic duplication (Fig. S2) and encode identical proteins similar to the *E. coli* SymE N-terminus. These are preceded by transcriptional regulators containing N-terminal helix-turn-helix (HTH) DNA binding domains (pfam HTH_3), which may serve as antitoxins, suggesting that these loci are hybrid type I/type II TA systems. No insertions were identified in *symE-1*, *symE-2* or their putative antitoxins, despite being expressed (Table 1). The lack of insertions in neighbouring genes, however, suggested that this region is essential for cell growth, that the transposon is not able to access it [58] or a part of this genomic island was lost following genome sequencing. The remaining *symE* genes [3–6] were predicted pseudogenes with none expressed under standard growth conditions and all tolerating transposon insertions (Table 1). In conclusion, there is evidence of expansion and inactivation of *symE* genes in *Serratia *, with one being similar to a previously defined SymE system and two potentially being part of novel type II loci.

### Type II systems are abundant in *Serratia*

The defining feature of type II TA systems is that toxin and antitoxin proteins interact to form non-toxic complexes (Fig. 1b) [59, 60]. Bioinformatic analysis predicted 19 type II TA systems of the HipAB, HicAB, HigAB, VapBC, ParDE and CcdAB families, and the RelBE/ParDE superfamily (Table 1). Combining TIS and RNA-seq, we determined that 8 of the 19 systems encoded toxins that were expressed at sufficient levels during growth in complex media to elicit toxicity when antitoxin production ceased (Table 1). Six of these TA loci resided in either genomic islands or prophages.

**Fig. 2.**
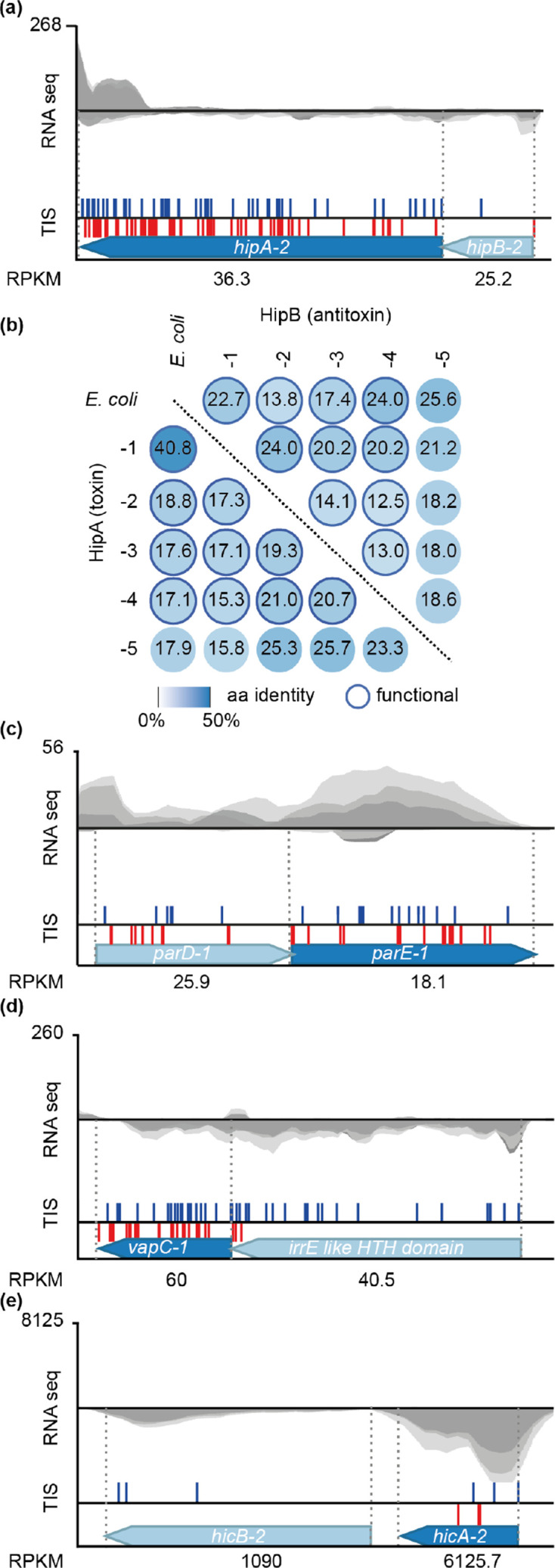
Type II systems are abundant in *Serratia *. (a) An example of transposon insertions (TIS, blue and red lines indicate insertions in the top or bottom strand, respectively), expression levels (*n*=3) as grey traces and quantified as RPKM, and organization of the genes encoding the HipAB-2 system. (b) Amino acid sequence similarity of the five *Serratia* HipAB systems and the *E. coli* K-12 HipAB across the entire length of the protein. Dark outer circles indicate a functional system. (c, d, e) Schematics of the non-functional *parDE* locus (c) and the functional (d) *vapBC-1* and (e) *hicAB-2* loci.

HipAB systems were first identified for their role in bacterial persistence [61]. The *E. coli* HipA toxin phosphorylates and deactivates glutamyl-tRNA-synthetase, resulting in bacteriostasis via the accumulation of uncharged tRNA^Glu^ [62]. Four HipAB systems were functional based on TIS data and two of these resided in genomic islands (Fig. 2a, b, Tables 1 and S5). RelE and ParE toxins have different mechanisms, but are grouped into the RelBE/ParDE superfamily based on common ancestry [63]. However, different antitoxin superfamilies can neutralize RelE and ParE toxins, helping to distinguish the RelBE from the ParDE family [50, 64]. Two of the five RelBE/ParDE superfamily systems identified were functional based on TIS and located in genomic islands (Table 1). One canonical ParDE system containing a ParD antitoxin was deemed non-functional due to TIS (Fig. 2c). A functional ‘mix and match’ system consisting of a VapC toxin and an IrrE-like HTH-containing antitoxin was identified (Fig. 2d), as was one of the two HicAB systems (Fig. 2e). The functional *vapBC* and *hicAB* loci reside in a genomic island and a prophage, respectively (Table 1). The remaining predicted systems whose function was not confirmed by TIS and RNA-seq (including the type V and VII) are described in more detail in Supplementary Results. In summary, *Serratia* contains a diverse array of 21 type II TA systems, and TIS led to the rapid validation of eight members of the HipAB, RelBE, ParDE, VapBC and HicAB systems as functional.

### *Serratia* contains four type IV systems

**Fig. 3.**
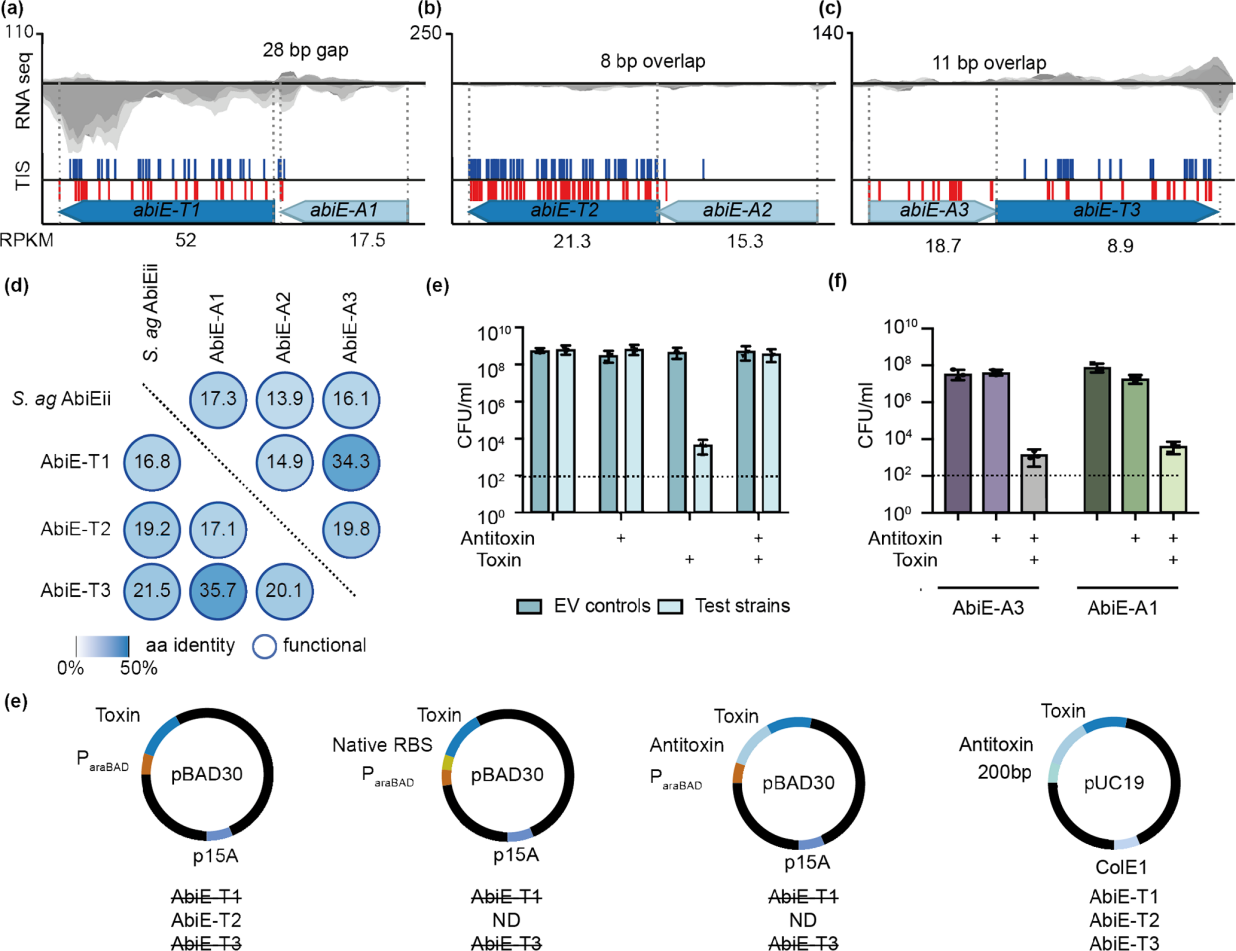
Three AbiE systems function as TAs in *Serratia.* (a, b, c) Transposon insertions (TIS, blue and red lines indicate insertions in the top or bottom strand, respectively), expression levels (*n*=3) as grey traces and quantified as RPKM, and genomic organization of the (a) *abiE-1*, (b) *abiE-2* and (c) *abiE-3* systems. (d) Protein identities (percent aa identity) of the AbiE systems compared to *S. agalactiae* (*S. ag*) AbiE. Antitoxin comparisons are shown in the lower triangle and toxin comparisons in the upper right. (e) Strategies employed for toxin cloning including the *araBAD* promoter to repress toxin transcription, the same *araBAD* promoter upstream of the native RBS, cloning the antitoxin and toxin together downstream of the *araBAD* promoter, and finally the antitoxin, toxin and 200 bp upstream of the antitoxin into pUC19. A strikethrough indicates only clones with mutated toxins were isolated. ND denotes the strategy was not attempted as a previous one had been successful. (f) Kill-recue assays with strains expressing empty vector (EV) or AbiE-A2 (pPF697) alone, AbiE-T2 (pPF703) alone, or both antitoxin and toxin together (pPF697+pPF703). Ara was used to induce toxin expression, and IPTG was used to induce antitoxin expression. Strains containing the plasmids were grown with glucose (toxin repression) to exponential phase. Cells were plated onto LBA+Ap+Km (no induction of plasmids), LBA+Ap+Km+IPTG (antitoxin induction), LBA+Ap+Km+Ara (toxin induction) and LBA+Ap+Km+IPTG+Ara (antitoxin and toxin induction). (g) Kill-rescue assays using AbiE-A1 (pPF699) and AbiE-A3 (pPF698) with AbiE-T2 (pPF703). Dotted lines show the limit of detection. Data shown are the mean and error bars represent the sem (*n*=3).

Type IV systems consist of two protein components, but there is no stable TA interaction [7, 65]. Antitoxins of type IV systems inhibit toxicity either by acting directly on the target or through modification, and inactivation, of the toxin [65, 66] (Fig. 1b). Four type IV TA systems were identified in *Serratia,* three of which were deemed functional. The three functional type IV systems belong to the widespread AbiE family, found in >3200 sequenced species [7, 8, 11]. Multiple members of the AbiE family have also been shown to provide phage resistance [8, 11]. Expression of all three AbiE systems was detected by RNA-seq and they were deemed functional by TIS (Fig. 3a–c, Tables 1 and S7). The toxin AbiEii belongs to the DNA polymerase β (DNA pol β) nucleotidyltransferase (NTase) superfamily, which transfers nucleotides to substrates such as DNA, RNA, proteins or small molecules [67, 68]. The three AbiE toxins had low sequence identity to *S. agalactiae* AbiEii [7] (Fig. 3d), but maintained the protein fold and conserved motifs necessary for toxicity [7] (Fig. S3). Antitoxins AbiE-A2 and AbiE-A3 are HTH-containing transcriptional regulators of the AbiE family, despite belonging to different Pfam clans [AbiEi_3 (CL0578) and AbiEi_4 (CL0123), respectively]. AbiE-A1 contains a DUF4095 domain, akin to AbiEi from *S. agalactiae* that has previously been shown to be a transcriptional regulator [7, 69]. An AbiE toxin homologue was recently shown to add pyrimidines to the 3′-CCA acceptor stem of specific tRNAs in *Mycobacterium tuberculosis *; however, it was unknown whether other AbiE toxins function similarly. Therefore, we further analysed the *Serratia* AbiE systems.

### Type IV AbiE systems are functional in *Serratia*

To confirm the three *Serratia* AbiE systems function as TA pairs and to validate the TIS results, we attempted to clone these systems in *E. coli* (Fig. 3e). All antitoxin clones were obtained, but only the *abiE-T2* toxin could be cloned despite using the tightly controlled *araBAD* promoter [47]. Clones of *abiE-T1* and *abiE-T3* always contained mutations, including when the cognate antitoxins were pre-expressed in the competent *E. coli* cloning cells prior to transformation. However, we were able to clone all *abiE* systems in their native operon contexts with their antitoxins and ~200 bp 5′ of the antitoxin start codon included. The *abiE* operon from *S. agalactiae* is subject to autoregulation by AbiEi [7, 69]; therefore, these cloning results indicate that autoregulation is also likely important for control of the three *Serratia* AbiE loci.

Upon AbiE-T2 overexpression in *E. coli *, a 10^6^-fold reduction in viable count was observed (Fig. 3f). Toxicity was counteracted by expression of the AbiE-A2 antitoxin, which confirmed that AbiE-2 functions as a TA module (Fig. 3f). There was no apparent cross-talk for AbiE-T2 neutralization between the AbiE antitoxins, as expression of AbiE-A1 or AbiE-A3 did not suppress AbiE-T2 toxicity (Fig. 3g). In agreement, AbiE homologues in *M. tuberculosis* were also independent of each other [28]. This finding supports the TIS data which showed that all three AbiE antitoxin genes were essential. In summary, the ability to clone the antitoxins but not the toxins alone (except AbiE-T2 when tightly repressed) support the TIS data and demonstrate that these three AbiE systems are functional.

### AbiE toxin is a predicted tRNA nucleotidyltransferase

A recent study of an AbiEii homologue in *M. tuberculosis *, MenT_3_, showed that toxicity can be partially mitigated by the overexpression of RNase PH, an enzyme important for tRNA processing, and that the toxin transfers pyrimidines to the 3′-CCA acceptor stem of select tRNAs [28]. To determine whether this mode of action is widespread among AbiE homologues, we sought to identify RNase PH, or any other protein-encoding gene that attenuated toxicity. We screened the ASKA library containing plasmids overexpressing *E. coli* protein-encoding genes [70] for proteins that mitigated AbiEii toxicity. However, the only reproducible hits we obtained were Ara efflux genes, which prevent AbiEii induction, but we did not observe protection by RNase PH expression. MenT_3_ is less toxic in *E. coli* than in mycobacteria, and is unable to target tRNAs without a 3′-CCA [28]. The inability to detect RNase PH from the ASKA library is likely due to differences in toxicity and tRNA processing between the original host.

**Fig. 4.**
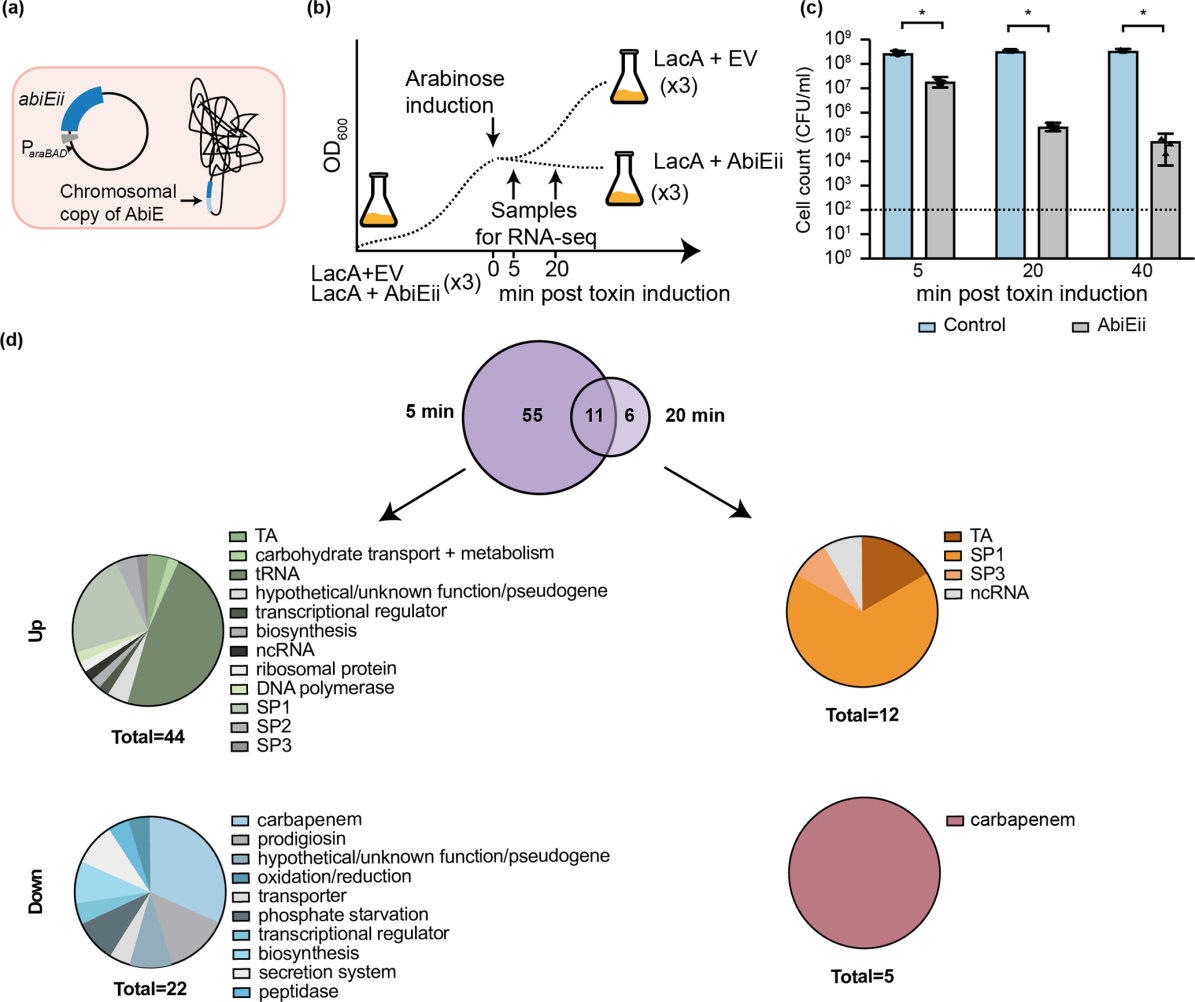
Induction of toxin expression results in differentially expressed genes. (a) Schematic of experimental set up showing the plasmid used for overexpression and the chromosomal copy of AbiE (both the toxin and the antitoxin). (b, c) Schematic of experimental set up (b) and changes in c.f.u. (c) at 5, 20 and 40 min post-toxin induction. Dotted lines show the limit of detection. Data shown are the mean and error bars represent the sem (*n*=3). (d) Genes that show significant differential expression at 5 and 20 min post-toxin induction. Genes are grouped into upregulation or downregulation, and divided into functional groups using gene set enrichment analysis. Note that in certain cases (e.g. genes found in prophages, the AbiE system, carbapenem and prodigiosin), location was used for grouping as opposed to the proposed function of the gene. ncRNA, Non-coding RNA.

To identify RNA targets and examine the transcriptome response to AbiE-T2 activity, we performed RNA-seq of a toxin induction time-course in *Serratia* (Fig. 4a, b). Overexpression of AbiE-T2 caused a severe growth impairment and reduction in viable cell count, despite the chromosomal cognate antitoxin (Fig. 4a, c). A total of 72 genes were differentially expressed upon toxin induction, with 55 unique to 5 min, and 6 unique to 20 min (Fig. 4c, Tables S7 and S8). The most differentially expressed genes were *abiE-A2* and *abiE-T2*, both with large increases (Fig. 4d, Tables S7 and S8). The *abiE-T2* increase is unsurprising, since this was expressed from the plasmid. However, the elevated *abiE-A2* is the result of chromosomal expression and suggests that AbiEii influences AbiEi-mediated autoregulation of the AbiE operon [69] (Fig. 4b).

**Fig. 5.**
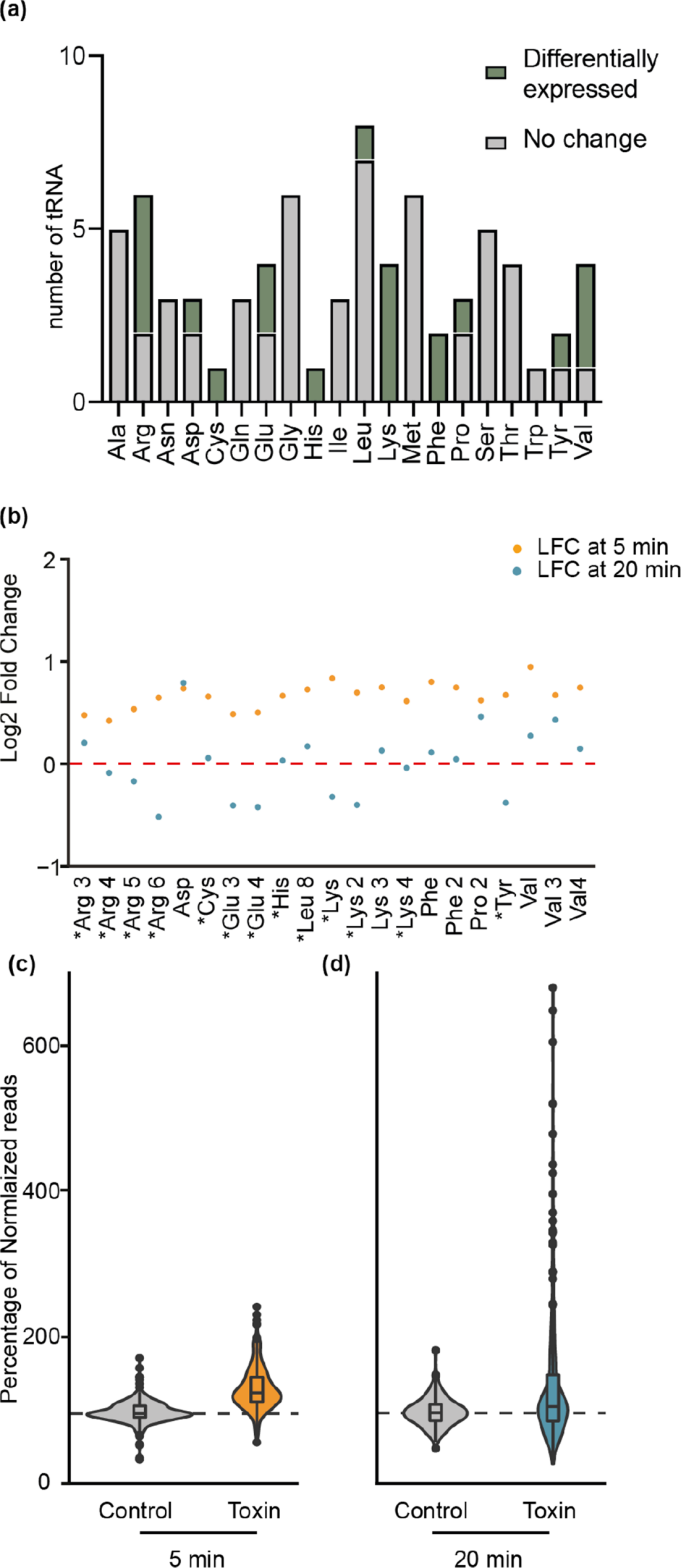
Total and differentially expressed tRNAs in *Serratia* upon AbiE-T2 toxin expression. (a) Proportions of tRNAs in *Serratia* that are impacted upon toxin expression. Light grey bars represent non-differentially expressed tRNAs, whilst green bars show differentially expressed tRNAs. (b) The log2 fold change (LFC) of reads mapping to differentially expressed tRNAs at 5 min (orange dots) and at 20 min (blue dots). The red dashed line represents expression levels in the absence of toxin induction (vector control). An asterisk denotes a significant difference (*P*_adj_ <0.1) between 5 and 20 min samples. (c, d) Percentage of normalized reads between the 5 min control and toxin strains (c), and the 20 min control and toxin strains (d).

In support of the recent *in vitro M. tuberculosis* AbiE data [28], the major category of genes differentially expressed *in vivo* upon toxin induction was tRNAs (Fig. 4d, Table S7). *Serratia* has 74 tRNAs, of which 21 displayed significant changes 5 min after toxin induction (Fig. 4d, Table S7). These tRNAs de-code 11 amino acids, and for some amino acids, all tRNAs were affected [Lys (×4), Phe (×2), Cys (×1) and His (×1)] (Fig. 5a). No tRNAs decoding Ser, while only one decoding Leu, were identified, despite the preference for these tRNAs by the *M. tuberculosis* homologue MenT_3_ [28]. Of the 21 tRNAs significantly elevated at 5 min after toxin induction, all but one decreased by 20 min (Fig. 5b). However, there was no clear trend for the expression of tRNAs at 20 min (Fig. S4). Thus, we examined the differences between total tRNA reads in each sample. At 5 min post-toxin induction, the median number of reads for tRNAs was higher than for the control (Fig. 5c). At 20 min post-toxin induction, the dispersion of reads was much greater than that of the control, whilst the median appeared unchanged compared to the empty vector control (Fig. 5d). While differences in toxin to antitoxin ratio may contribute to the variation in tRNA expression between the samples following toxin induction (Fig. S5), the changes in tRNA expression are likely to be underestimated due to the RNA-seq method. Overall, the increased levels of AbiE-T2 detected result in tRNA dysregulation. This dysregulation differs from the stringent response, where tRNA levels decrease from as early as 5 min after induction [71]. Based upon sequence alignments, sequence relatedness and genomic context, it was not possible to determine whether there was specificity in the tRNA targeting, as has been noted for other TA systems that perturb tRNA functioning (Figs S6 and S7) [28, 72]. In summary, the AbiE toxin belongs to the nucleotidyltransferase family, in our experiments was unable to be counteracted by a single overexpressed protein and affected tRNA abundance. Combining these results with the recent *M. tuberculosis* data [28], we propose that tRNAs are the target of AbiE-T2 in *Serratia* and hypothesize that AbiEii-like proteins in general may function through tRNA targeting.

## Discussion

Here, we developed a high-throughput functional genomics approach to test the activity of 32 TA systems identified in *Serratia* sp. ATCC 39006, confirming eight type II and three type IV TA systems as functional. Furthermore, we showed that of the seven type I TA systems, four are likely pseudogenes. We demonstrated that TIS is a quick and unbiased method to validate TA system function within a native host bacterium under physiological expression levels and defined growth conditions. Recently, increased transcription of TA systems under stress conditions was shown not to be an accurate proxy for TA system activation, as had been previously accepted in the field [54]. We propose that mimicking the loss of the antitoxin (by transposon disruption) provides a good method to determine whether triggering the release of the normal physiological levels of the toxin induces growth inhibition. Despite these benefits, and not being reliant on cloning individual systems, there are some caveats. For example, antitoxin redundancy (or cross-talk) may obscure essential antitoxins and TIS does not confirm other systems as non-functional, since they may be active under differing conditions than those tested. However, TIS could be readily performed under a variety of conditions to identify essential genes (e.g. antitoxins) [53].

We identified three functional AbiE type IV TA systems in *Serratia *, which are widespread and yet poorly characterized. Recently, the antitoxin of an AbiE system was shown to inhibit toxicity through phosphorylation of the toxin in *M. tuberculosis* [66]. The toxin residue (S78) that is phosphorylated is within the conserved motif I and is conserved in *Serratia* AbiE-T2 (S46) (Fig. S3). However, the residues identified as important for the phosphorylation activity by the antitoxin are less clear in the *Serratia* AbiE antitoxins (Fig. S3). It has been demonstrated that AbiE toxins belong to the nucleotidyltransferase family and can bind specific nucleotides [7, 66]. A recent study showed that an AbiEii homologue, MenT_3_, adds pyrimidines to the acceptor stem of tRNAs that possess a 3′-CCA. Curiously, MenT_3_ is pyrimidine specific, whilst previous studies have shown that AbiEii from *S. agalactiae* has a preference for GTP, which may highlight that various AbiE/DUF1814 toxins have different specificities. We propose that AbiE-T2 transfers nucleotide(s) to tRNA(s), leading to tRNA(s) that fail to be charged, and/or have altered stability - akin to MenT_3_. This activity impairs translation and causes bacteriostasis, but is reversible upon antitoxin expression [7]. This model of AbiE toxins as tRNA nucleotidyltransferases is supported by two additional lines of evidence. Firstly, we were unable to suppress the toxin by overexpression of potential protein targets. The second line of evidence is that RNA-seq revealed that tRNAs were significantly affected after toxin induction. Since bacterial tRNAs are highly stable unless compromised in quality [71], these rapid fluctuations in the tRNA pool support that they are the direct target of the AbiE toxin. In agreement, two recent studies have shown that translation is affected by the expression of mycobacterial AbiE homologues [28, 66]. However, we predict that there are important differences between such homologues, including the nucleotides involved in the reaction (pyrimidines versus purines/GTP) [7, 28], and the targeted tRNAs. This suggests that while tRNA targeting to halt translation might be widespread and conserved among AbiEii-like proteins, differences are likely to occur both between organisms and multiple AbiE systems present in a single organism (e.g. *Serratia *). Definitive proof of a tRNA nucleotidyltransferase activity of the *Serratia* AbiE toxins will require further biochemical experiments.

Targeting of translation processes by toxins is widespread, with VapC, TacT/AtaT, MazF, HipA, Doc and RelE all impairing translation [72]. Some are toxic through their direct targeting of tRNAs. For example, the VapC toxin cleaves the initiator *N*-formyl-methionyl-tRNA within the anticodon stem loop, while the AtaT toxin acetylates this same tRNA [73, 74]. TacT acetylation is less specific for a particular tRNA and affects tRNA^Leu^, tRNA^Gly^ and tRNA^Ile^ [75, 76]. MazF cleaves UUU sites in the anticodon loop of tRNA^Lys^, and the D loop of tRNA^Pro^, among other substrates [77–79]. How toxins distinguish between tRNA species is not known; however, it is likely that both tRNA sequence and structure are important [72]. MenT_3_ was shown to be highly specific [28]. In accordance, we observed that not all tRNAs were equally affected by AbiE-T2 overexpression, suggesting AbiE-T2 is also specific for certain tRNA(s) and that these differ from those targeted by MenT3. This is consistent with the lack of cross-talk between the three *Serratia* AbiE systems, which may each have different tRNA target(s). Modifying a small number of tRNAs would result in a halt in protein synthesis by strongly affecting codon usage, and may result in alterations to distinct parts of the proteome [50].

As Abi/TA systems function as innate immune systems that protect bacteria from phage infection, it is inevitable that phages have developed the ability to overcome these systems [1]. Interestingly, many phages express their own tRNAs [80], which likely assist in phage replication. Phage tRNAs might act as decoys to inactivate and sequester toxins of TA systems. Alternatively, phage tRNAs might provide a backup for the phage to compensate for host tRNAs modified by the toxin, or be a variant that is resistant to modification.

In conclusion, we have demonstrated the utility of TIS to predict TA system function in native hosts in a genome-wide high-throughput manner. This has rapidly expanded our knowledge of the innate genome defence repertoire in *Serratia* and advanced our mechanistic understanding of type IV AbiE systems. Analysis of the role these innate systems alongside CRISPR-Cas immunity will be essential to elucidate the combined bacterial immune response against the phages that infect them.

## Supplementary Data

Supplementary material 1
