# Supplementary material for: Functional genomics reveals the toxin–antitoxin repertoire and AbiE activity in Serratia
Source: Microb Genom. 2020 Oct 19;6(11):mgen000458. doi: 10.1099/mgen.0.000458 (PMC7725324; doi:10.1099/mgen.0.000458)
Supplement: Supplementary material 1 [file mgen-6-458-s001.pdf]

Functional genomics reveals the toxin-antitoxin repertoire and AbiE activity in *Serratia*

Hannah G. Hampton, Leah M. Smith, Shaun Ferguson, Sean Meaden, Simon A. Jackson and Peter C. Fineran

Supplementary material

Supplementary Results

Type I, II, IV, V and VII systems in *Serratia*

In *Serratia*, the seven *symE* genes (SymE_1-7 based on genome position) were highly variable in length and predicted to encode proteins from 29 to 114 aa in length. We predict that *symE-3*and *symE-4* genes are most likely pseudogenes. Firstly, the predicted proteins are only 53 and 29 aa, respectively, map to internal parts of *E. coli* SymE and have rare initiation codons (CTG). Secondly, they are nearby multiple pseudogenes within a region that appears in a process of degeneration. In agreement, these genes were not expressed (Table 1). Automated annotation of SymE-5 and SymE-6 toxins (both 84 aa) indicated ATT (Ile) start codons. We manually identified ATG start codons for both genes that led to 111 aa proteins (96.4% id) and aligned along the full length of *E. coli* SymE. A Type II *parDE* system is present between the *symE_5* and *symE_6* genes and this region has evidence of recombination and pseudogenesis. The near-identical nature of these *symE* genes, suggests they arose by duplication, potentially during insertion of the *parDE* system. Due to the prediction of *symE1-6* being pseudogenes and

In addition to the Type II systems that were shown to be functional using TIS and RNA-seq, a number of other Type II systems are present in *Serratia* and are discussed below. Firstly, two were identified as orphan toxins or antitoxins, that is that their cognate partner could not be identified. VapC-2 is an orphan toxin, that may be neutralised by the HTH domain protein present in VapBC-1, or alternatively may no longer function as a toxin. Phd was identified as an orphan antitoxin, with no Doc (or other) toxin nearby. The *Serratia* Phd-1 is a 38 aa protein that aligns within the N-terminus of the 73 aa *E. coli* Phd suggesting it is truncated. In *E. coli,* the N-terminus is a dimerization and DNA binding domain involved in autoregulation, while C-terminal domain is involved in toxin neutralization (1). The *phd-1* locus is immediately upstream of the S20 subunit gene of the 30S ribosome, which is curiously the target of P1 Doc. The absence of a Doc toxin, and the essentiality of Phd could indicate it acts as an antitoxin for another system, however the absence of a toxin neutralizing domain suggests that this is unlikely. Rather Phd-1 likely affecting the transcription of the 30S ribosomal protein as no insertions are tolerated between open reading frame and the 30S ribosomal protein. Several systems that were not described as validated may be the result of functional redundancy, whereby an antitoxin from the same family that lies elsewhere in the genome is able to provide the antitoxic function. This applies to 7 systems, namely HipAB-5, RelBE/ParDE-1, -2 and -5, HicAB-1, and HigAB-1 and 2. The remaining two systems a *parDE-1* and a *ccdAB-1* tolerated insertions and were expressed suggesting that these systems are not functional in the conditions tested.

The type IV system, CbtA-YeeU, in *E. coli* inhibits cytoskeleton polymerization and thus cell division (2). Using TIS, this system was deemed non-functional in *Serratia* (Table 1). An additional proposed Type IV system from *E. coli* (CptA-YgfY) was present in *Serratia* (*sdhE-ygfX*), but was previously demonstrated to have no TA function. In agreement, *sdhE-ygfX* was not found during TA system identification and was not included in our analyses (3,4).

GhoST is the only described type V system and its function relies on a Type II toxin for activation(5). Here the antitoxin, GhoS, is degraded by the Type II toxin MqsR under conditions of stress. This results in translation of GhoT, a small hydrophobic protein. In the absence of stress, the GhoS antitoxin is an RNase that cleaves *ghoT* mRNA(5). Interestingly, *Serratia* contains an orphan GhoT toxin (39% id to *E. coli* GhoT). Another RNase within the cell may be able to compensate for the absence of any detectable GhoS. The level of expression of *ghoT1* was higher than expected for the toxin with no detectable antitoxin and unsurprisingly, tolerated insertions (Table 1). An oxygen dependent type VII system has recently been described whereby through transient interaction, the antitoxin promotes the spontaneous oxidation of and thus destabilizes the toxin (6). A single type VII system, TomB-Hha was identified. This system was expressed but was able to tolerate insertions.

Supplementary Methods

Validation of genome duplication

PacBio reads were mapped to the *Serratia* genome using minmap2 (version 2.1)(7) with the PacBio presets option (map-pb). Alignments were sorted an indexed using samtools (version 1.9)(8) with coverage and mapping information extracted with bedtools (version 2.29.2)(9) then visualised in R (version 3.6.2)(10).

Supplementary Figures

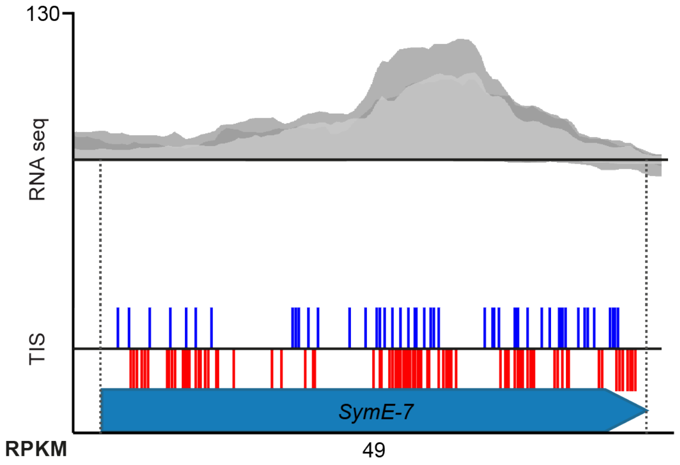

Figure S1: SymE-7 tolerates insertions and is expressed in *Serratia.* Transposon insertions (TIS, blue and red lines indicate insertions in the top or bottom strand, respectively), expression levels (*n*=3) as the grey traces and quantified as RPKM, and genomic organisation of *symE-7*.

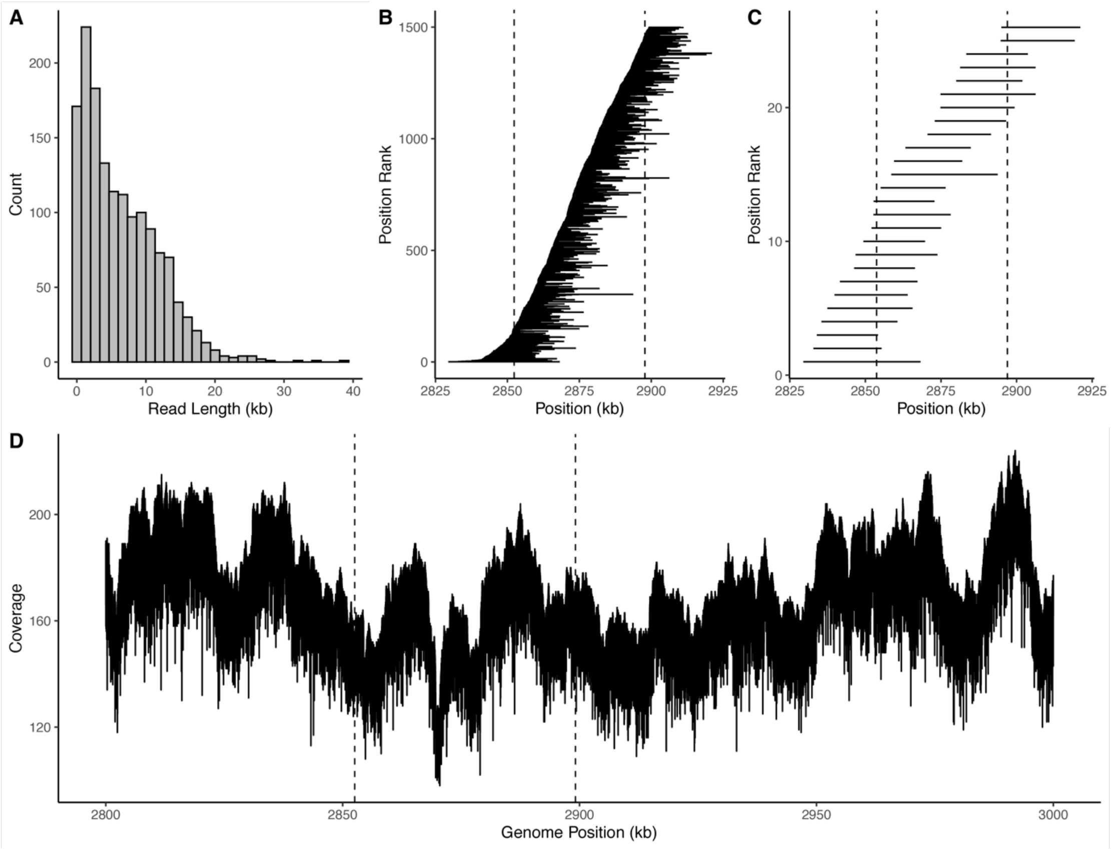

Figure S2: The SymE genomic duplication is not an artefact of genome assembly as the read depth does not significantly decrease across the duplication region. (A) Histogram of read lengths of PacBio reads that map to the region from 2863918 bp - 287719 bp of the *Serratia* genome that contains *symE-1* and *symE-2*. (**B**) All PacBio reads mapping to the region of interest and **(C)** PacBio reads longer than 20kb that map to the region of interest dashed lines denote proposed boundaries of duplication. **(D)** Coverage across the region of interest and flanking regions with the Illumina sequencing. Mean coverage for the whole genome with PacBio reads is 204, and Illumina reads is 465.4.

A

```
AbiE-A2           MSSKLNWLLQKTAPGSLILQSWLTKNGISPSLANKYM-HSDWLQKLRAGVYYVRSGRDPQW 59
AbiE-A1           -------------------------------MAKSDELLNVFAKAVRAGGGVHSATELAF 29
AbiE-A3           -------------------------------MQQTTA-IQRLSELDQQGRYVFSQRDLEK 28
AbiEi Sag         -------------------------------MSKKEILLDFIEK---NNGIVTN-KD-CK 24
MenA3             ----------------MCAKPYLIDT-IA-HMAIWDRLVEVAAE---QHGYVTT-RD-AR 37
                                                 :       .   :       * .  :

                                     Helix 3
AbiE-A2           SDAVLCLQNQLGISVHLAGLTSLTYQGRSHYLHLTRQAIWLCVE-----DKASLPKWFKE 114
AbiE-A1           MLGVPCGPAF------RKLLADSVKKGL---LRRVVKGFYESMITPPE-PETAIYKIIKK 79
AbiE-A3           IFSEDSKRTL------HAGLERLVRQNI---LERAARGVYLFALSHHKKGPDTLELIAKT 79
AbiEi Sag         ALGIP-----------TIYLTRLEKEGI---IFRVEKGIFLTQN---GDYDE-YYFFQYR 66
MenA3             DIGVD-----------PVQLRLLAGRGR---LERVGRGVYRVPVLPRGEHDDLAAAVSWT 83
                    .                *     ..    :  . :..:

AbiE-A2           FPNVEWLLLSNQKLNMHDEKYLTEVEIKGGRL-KASVPELAAYEIANAVPGSLSFEHAAE 173
AbiE-A1           LRSGVLNYISLESQLSYTG---DISQIVMGRITVVTKGRSGCF---DTPYGV-------- 125
AbiE-A3           LRRGEYNYVSLESALSSYG---VISQIPMDRLTVMTTGRKGEY---KTHYGT-------- 125
AbiEi Sag         FPKAIFSYISALYLQQFTD---EIPQYF-----DVTVPRGYRF---NTPPAN-------- 107
MenA3             LGRGVISHESALALHALAD---VNPSRI-----HLTVPRNNHP---RAAGGE-------- 124
                  :        *               .         :  .        :  .

AbiE-A2           LFQGLVNLSPRKVELLLQSSRAVQTNRLYLFLADH------YAHAWVKRIDKANIDLGAG 227
AbiE-A1           ------------IEFTHTKRPVEQIAPNLYYDPDIKMYRAFKEQAIAD------------ 161
AbiE-A3           ------------IEFTHTKRPVSEIIQSFISIN-RPLRMATKQAAWRD------------ 160
AbiEi Sag         LNIHFVSKEYSELGMTTVPTPMGNNVRVYDFE-----------RIICD------------ 144
MenA3             LYR----VHRRDLQAAHVTSVDG--IPVTTVA-----------RTIKD------------ 155
                              :                                  .

AbiE-A2           KRQIVSGGKLDPKYQITVPESFVSKGISHG------------------------------ 257
AbiE-A1           --LKHCRRNLH----MLES----------------------------------------- 174
AbiE-A3           --QKRVGRNTH----LVQKEMLNED----------------------------------- 179
AbiEi Sag         --FVIHREK-------IDSELFVKTLQSYGNYPKKNLAKLY----EYATKMNTLEKVKQT 191
MenA3             --CVKTGTD------PYQLRAAIERAEAEGTLRRGSAAELRAALDETTAGLRARPKRASA 207
                          .

AbiE-A2           ----- 257
AbiE-A1           ----- 174
AbiE-A3           ----- 179
AbiEi Sag         LEVLI 196
MenA3             ----- 207
```

B

```
AbiE-T2           -------------------------------MDKHSPYYRQV---ELLLSALPVVASERC 26
AbiEii Sag        ------MNKAKLTALCHKISK------NTGLTFNSVMTYYFLEV---ILKKLSQSSYSNH 45
MenT3             MTKPYSSPPTNLRSLRDRLTQVA---ERQGVVFGRLQRHVAMIVVAQFAATLTDDTGAPL 57
AbiE-T1           ------MLK---QQIRQIVQSNPDYAAVTPVIEKEILHHDIMDV-------LIKQGVMQR 44
AbiE-T3           ----MKIDPADFNALVAKAMQQAEVAAMRPVIEKELLHYDILYC-------LDQAGLLNE 49
                                                        :  :         *

                   Motif I          Motif II
AbiE-T2           FALKGGTAINLFVRD-FPRLSVDIDLAYIPLESRDVALPNVRAALTRIAEIQQQ-QAGIS 84
AbiEii Sag        YIFKGGFLLSNVIGV-ESRSTVDIDFLFHQ----I-TLSE-ETVKQQLKEILADSEEGIS 98
MenT3             LLVKGGSSLELRRGIPDSRTSKDFDTVARR----DIELIH-----EQ------------- 95
AbiE-T1           LTFIGGTSLRMCYNS--SRLSEDLDFNGGH----DFKPSEFEGLESEIQTYIQN-KYETE 97
AbiE-T3           LVFQGGTSLRLCHGG--NRFSEDLDFAGGV----DFSSARLAEMKTCIEDYIGK-RYGLE 102
                    . **  :         * : *:*              .

AbiE-T2           AVLQTN---------------NPDEMRIIVSSP------EAQIK---------------- 107
AbiEii Sag        FVIQSITTIKESDD--------YGGYRATISC--QLENIKQVIH---------------- 132
MenT3             --------------LADAGETGWEGFTAIFTAPEEIDVPGMPVKPRRFTAKLSYRGRAFA 141
AbiE-T1           VWVNKPSGEQQG------DTVSWK--ISIVKEPNRPDLPRQKMH---------------- 133
AbiE-T3           VTVKEPASLKEEKEYAELRIDKWQ--INITTAPGQKDMPRQKIK---------------- 144
                                              .           ::

                                                             Motif III
AbiE-T2           ---IEVSPVTRGTLYPPEERDVVEAVDDEFGFATIQVVSLADLYGGKLCAAL----DRQH 160
AbiEii Sag        ---LDIATG--DVVTPQPITYDYKAIFDE-DNFPIIAYTIETILAEKLQTIYSRNFLNSR 186
MenT3             TVPIEVSSVEAGN------ADQFDTLTSDALG---------------LVGVPAAVA-V-- 177
AbiE-T1           ---IDVCAIPSFDIEKRPLINHYDIVVP-TEGILVPVQSLQETLADKLIALAYRAR-RIK 188
AbiE-T3           ---LEVANVPAYTKEALPLRNSYDFLPDGYEDTLVFTETLDEVMADKLISLPATQR-YIR 200
                     :::.                . :                     *

                  Motif IV
AbiE-T2           PRDFYDVKMLLETQGIDR------------H----IFNGFIT------------------ 186
AbiEii Sag        SKDFYDVYILS--------------KLKK---KDIDFNQLKN------------ACQRTF 217
MenT3             --PCMTIPWQIAQKLHAVTAVLEEPKVNDRAHDLVDLQLLEGLLLDADLMPTRSACIAIF 235
AbiE-T1           PRDIWDIVWIKQRG-IDLSKILVEKKLNARHKQTDDFRQALSTSLA-KLMSEE-EVHNDF 245
AbiE-T3           HRDLWDLVWLQQQN-AKLKPELIRLKLAD--YKIPDYQQLLESRIA-S-LDEI-VASAAF 254
                        :                              .

AbiE-T2           -YLLGHPRP------------LSEVLNPRWKDISLPYA--------NEFNGMTFDAISLE 225
AbiEii Sag        SYRETELDFEKIIELLERFKS-DPTQNQQWQNYSKKYSYTKGISLANVLDEMIS--LI-- 272
MenT3             EARAQHPWP-------PRV-----ATLPHWP-----LIYA------GALEGLDHLELA-- 270
AbiE-T1           NMEMSRFIP-------KQIKERTLDNPEYWA-----YV-Q------GEVKAIAF-EL--- 282
AbiE-T3           HNEMKRFLP-------TNVYDRTLGQDKFKS-----FM-L------GTLKKMLT-ELH-- 292
                       .                                        . .. :    :

AbiE-T2           ELNAIPKLMVTALKAQFTQRDYDFLMSFKRGEPDWNLAPEDQIQHLPAVRWKLRNIGRIP 285
AbiEii Sag        ---------TIIIS--TDSD---------------------------------------- 281
MenT3             ---------RTVDA--AAQAVQRFVARIDRATKR-------------------------- 293
AbiE-T1           ------------LH--DCTPKNPFDMG--------------------------------- 295
AbiE-T3           ---------TQLFG--SSTPEPEFKL---------------------------------- 307

AbiE-T2           KDKHIQALAKLETVLAEWIE 305
AbiEii Sag        -------------------- 281
MenT3             -------------------- 293
AbiE-T1           -------------------- 295
AbiE-T3           -------------------- 307
```

Figure S3: The *Serratia* sp. ATCC 39006 AbiE homologues show conserved motifs with both *Streptococcus agalactiae* and *Mycobacterium tuberculosis* homologues. (A) A Clustal Omega alignment of the *Serratia* AbiEi and **(B)** AbiEii homologues compared to the *S. agalactiae* AbiEi and AbiEii and *M. tuberculosis* MenA3 and MenT3. Residues in red show conservation between all four AbiE homologues and are denoted by a *, : denotes conservation between residues with strongly similar properties, and . indicates conservation between residues with weakly similar proteins. The letters in bold in motif II of **(B)** highlight the conservation of the serine residue that is phosphorylated by the antitoxin in the TakA/TakT system.

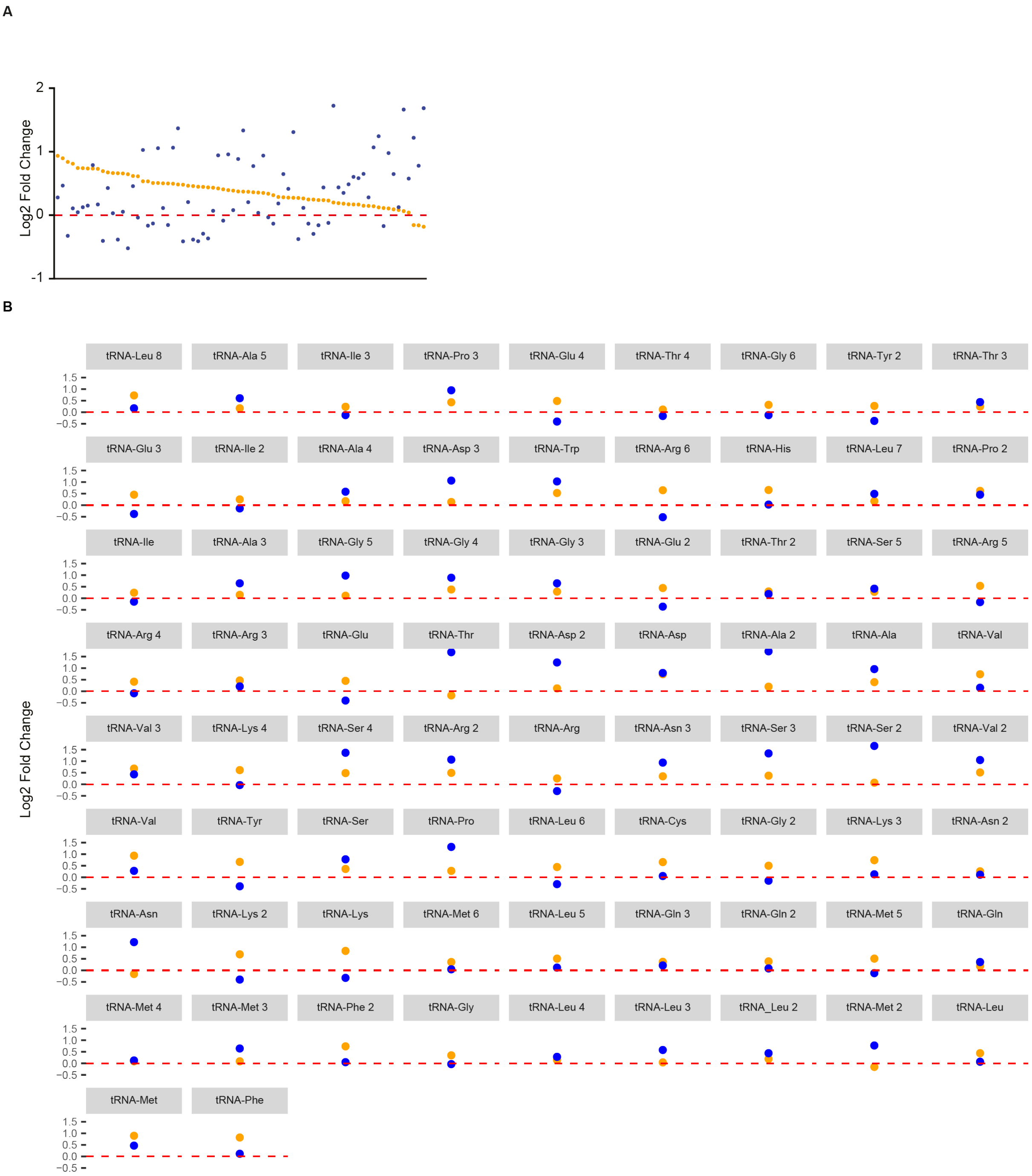

Figure S4: tRNA transcripts increase at 5 min, while there is no clear trend at 20 min post toxin induction. (A) Total tRNAs in *Serratia* at 5 min (orange dots) and at 20 min (blue dots) ordered by significance at 5 minutes. The red dashed line represents no change/the WT expression levels. (**B)** Each tRNA is plotted individually and labelled.

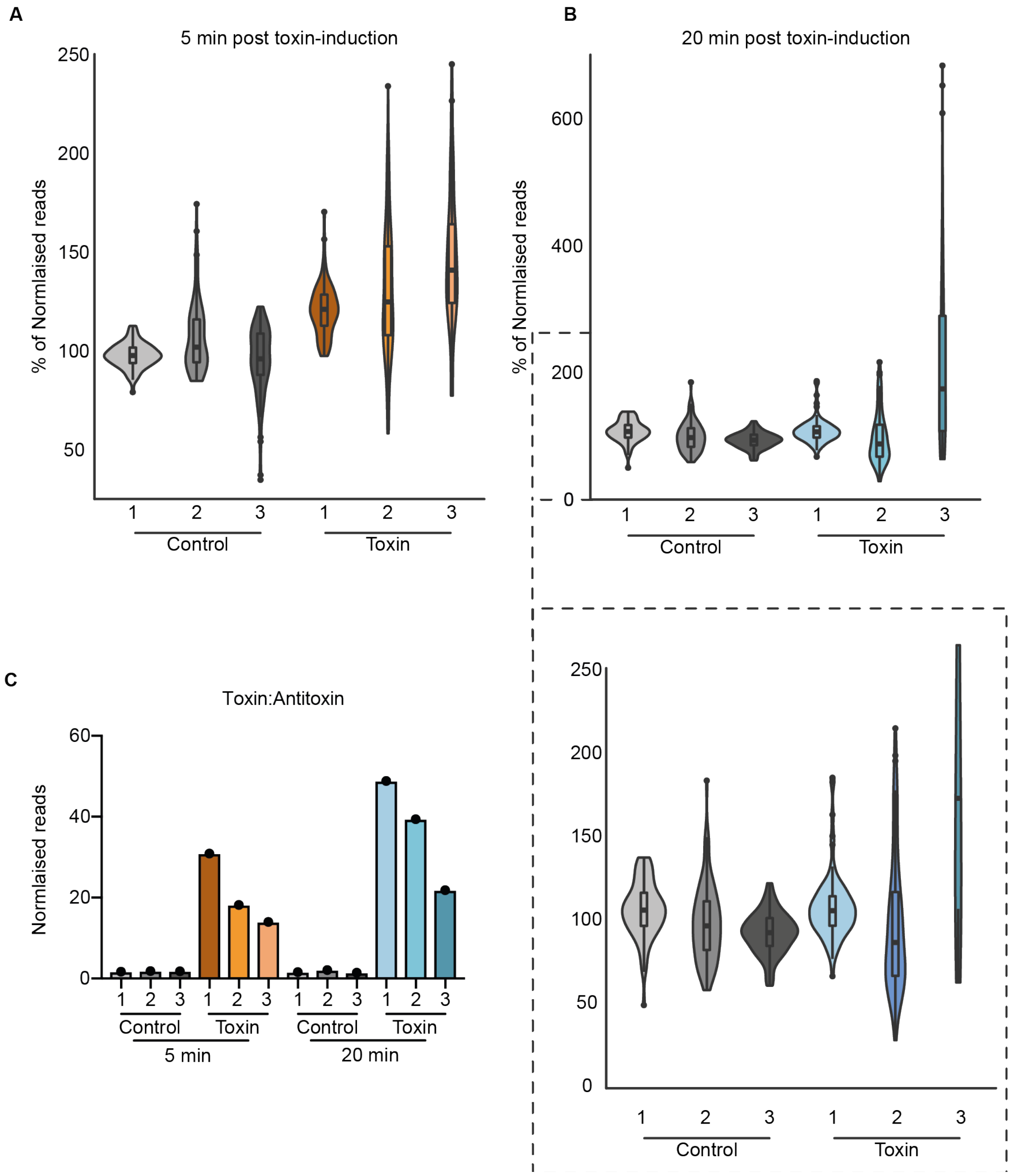

Figure S5: Dispersal from individual libraries used in RNA-sequencing show different dispersal. (A) The 5 min control and toxin samples normalised to the 5 min control and **(B)** the 20 min control and toxin samples normalised to the 20 min sample. Bottom panel of **(B)** zooms in on 0-250% of normalized reads. **(C)** The ratio of reads mapping to the toxin:antitoxin from the control and toxin-expressing samples. Note the large differences observed in the toxin induced strains.

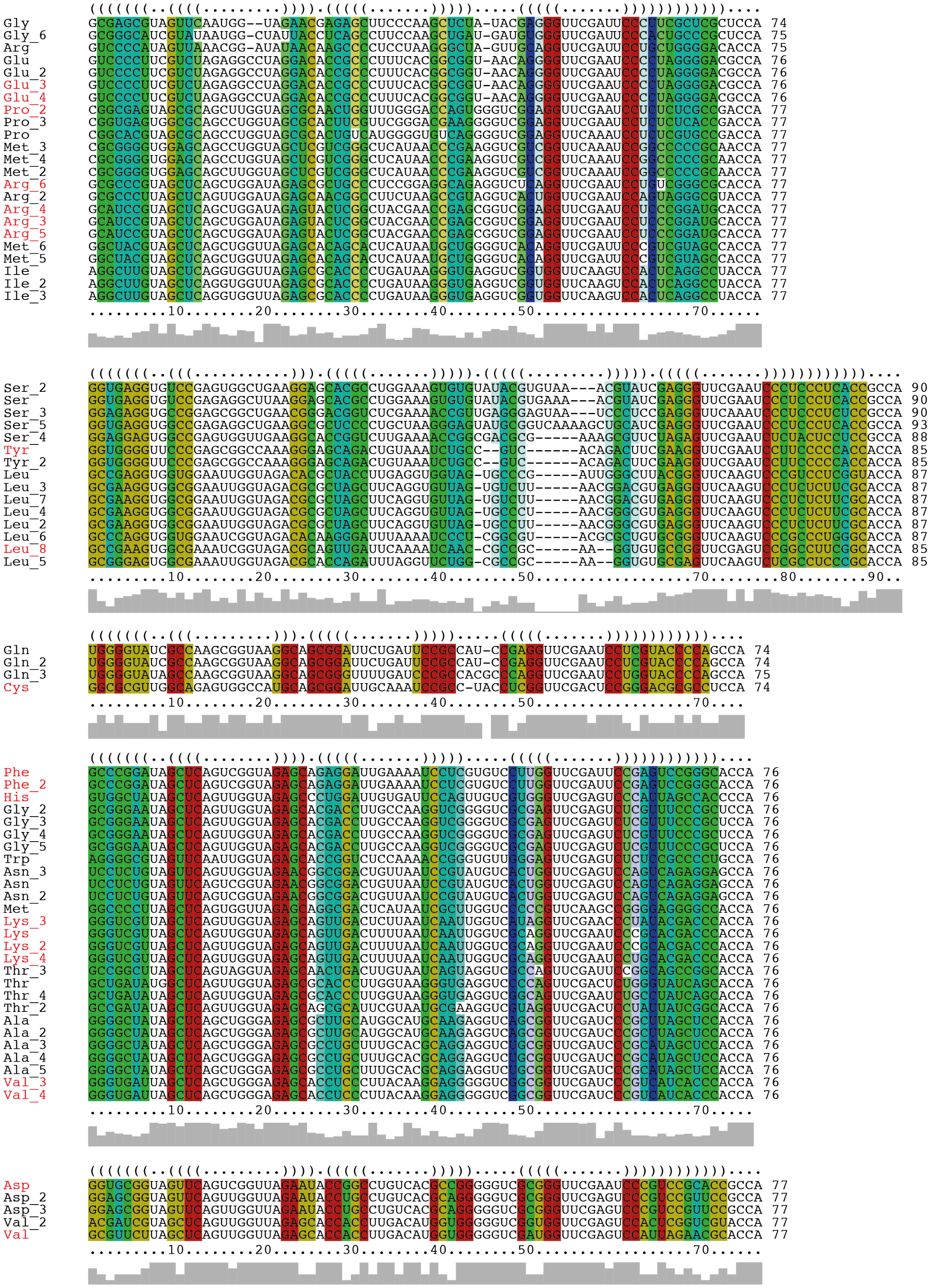

Figure S6: Alignment of *Serratia* tRNAs. The differentially expressed tRNAs at 5 min post-toxin induction (red) aligned to all tRNAs in *Serratia.* Aligned using LocARNA (11,12)

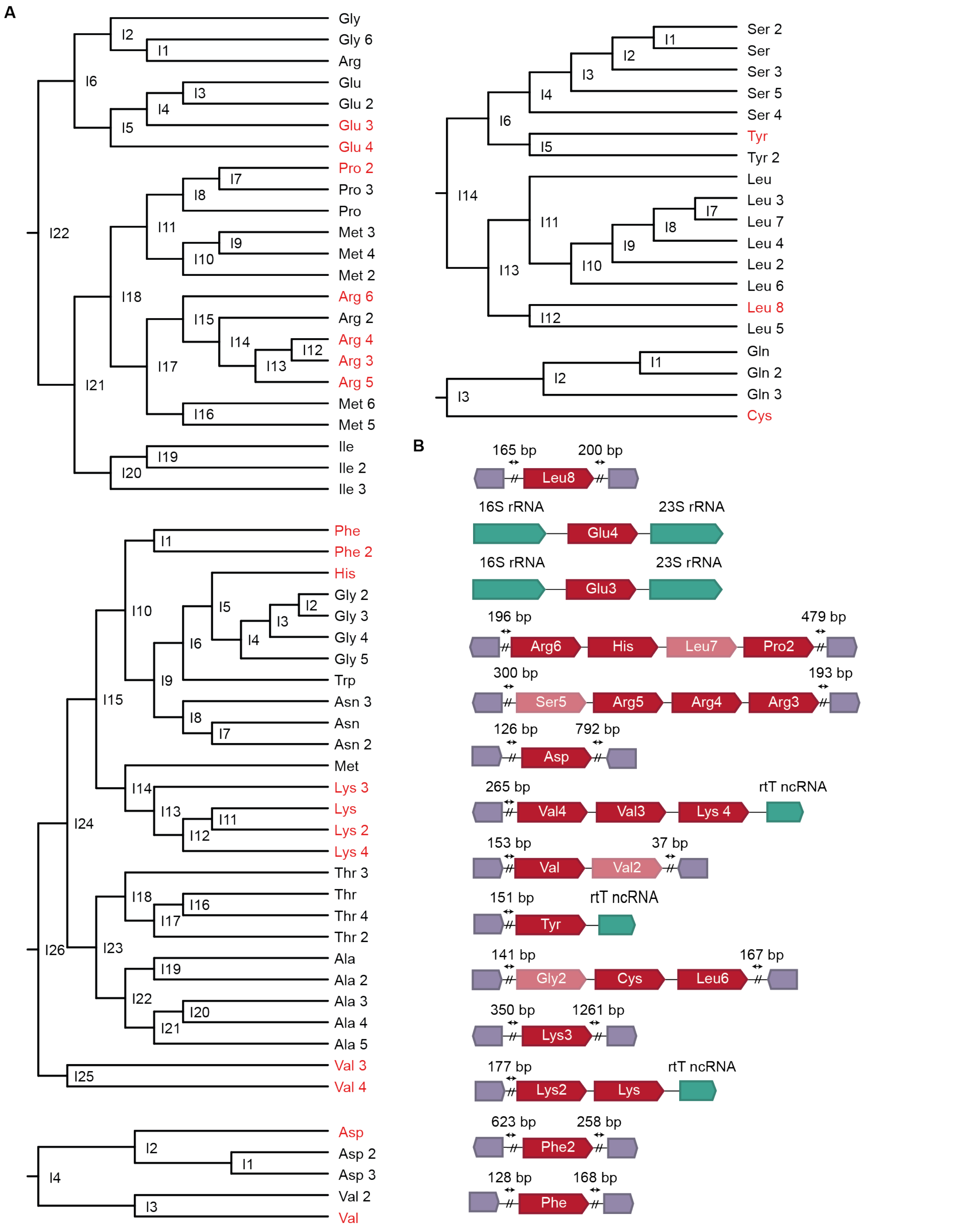

Figure S7: Similarities and locations of differentially expressed tRNAs. (A) Phylogenetic tree depicting relatedness of tRNAs in *Serratia,* based upon alignments from Figure S6 using LocARNA (11,12). Red indicates differentially expressed tRNAs at 5 min post toxin induction. **(B)** Schematics showing genomic locations of differentially expressed tRNAs. Differentially expressed (red) or non-differentially (pink) tRNAs, non-coding RNAs (green) and other coding sequences (purple).

Supplementary Tables

Table S1. Strains used in this study

**Strain**	**Genotype/Phenotype**	**Ref**
Escherichia coli		
E. coli DH5α		Gibco/BRL
E. coli ST18	Auxotrophic donor for biparental conjugation	(13)
Serratia derivatives		
Serratia sp. ATCC39006 WT		
Serratia sp. ATCC3906 LacA	Lac- mutant generated by EMS mutagenesis.	(14)
PCF396	LacA, Δ*pigA-O*	Smith unpublished.

Table S2. Plasmids used in this study

Plasmid	Genotype/Phenotype	Ref
pPF260	pTRB30-derivative, pMB1 ori, IPTG-inducible, RP4 *oriT*, Km^R	(15)
pUC19	ColE1 ori, Ap^R	(16)
pBAD30	Arabinose inducible expression vector, p15A ori, Ap^R	(17)
pRLD12	AbiEii from *S. agalactiae* in pBAD30	(18)
pPF697	AbiE-A2 in pPF260, Km^R	This study
pPF698	AbiE-A3 in pPF260, Km^R	This study
pPF699	AbiE-A1 in pPF260, Km^R	This study
pPF703	AbiE-T2 in pBAD30, Ap^R	This study
pAbiE-T1*	AbiE-T1 in pBAD30, Ap^R	This study
pAbiE-T3*	AbiE-T3 in pBAD30, Ap^R	This study
pAbiE-T1R*	AbiE-T1 in pBAD30 with native RBS, Ap^R	This study
pAbiE-T3R*	AbiE-T1 in pBAD30 with native RBS, Ap^R	This study
pAbiE1*	AbiE-1 operon in pBAD30, Ap^R	This study
pAbiE3*	AbiE-3 operon in pBAD30, Ap^R	This study
pPF802	AbiE-2 + 200 bp Upstream into pUC19	This study
pPF803	AbiE-1 + 200 bp Upstream into pUC19	This study
pPF805	AbiE-3 + 200 bp Upstream into pUC19	This study
pKRCPN2	R6K/ori, RP4/OriT, Tn-DS1028*uidA* Km, Transposase, Tc^R	(19)
pCA24N	pMB1 origin of replication (high copy number), IPTG-inducible (repressed by *lacI^q*). Cm^R	(20)
pCA24N + *ydeA*	pMB1 origin of replication (high copy number), IPTG-inducible (repressed by *lacI^q*), *ydeA* gene. Cm^R	(20)

*Denotes correct plasmid sequence was not obtained for these constructs as per Figure 3E.

Table S3. Oligonucleotides used in this study

Primer	Sequence (5'-3')	Notes[a]	Restriction Sites
PF209	TCGTCTTCACCTCGAGAAATC	F primer for amplifying ORF containing region of ASKA library plasmids	
PF210	GTCATTACTGGATCTATCAACAGG	R primer for amplifying ORF containing region of ASKA library plasmids	
PF796	ATAGAATTCAGGAGGAATATAATGGGTACT TCTGAGTTACTTAAGC	*flhDC* F primer for gDNA contamination check	EcoRI
PF797	GATCCCGGGTCAGACTGCGTGTTTTACTTG	*flhDC* R primer for gDNA contamination check	XmaI
PF1601	TTTGAATTCAGGAGAAATTAACTATGGGTG AATCTATGTCATCAAAG	AbiE-A2 F primer	EcoRI
PF1602	TTTGTCGACTTATCCATGCGAGATCCC	AbiE-A2 R primer	SalI
PF1603	TTTGAATTCAGGAGAAATTAACTATGGCAA AATCTGATGAGCTAC	AbiE-A1 F primer	EcoRI
PF1604	TTTGTCGACTTAGCTCTCCAGCATATGTAG ATTG	AbiE-A1 R primer	SalI
PF1605	TTTGTCGACAGGAGAAATTAACTATGCAAC AGACCACAGCG	AbiE-A3 F primer	SalI
PF1606	TTTCAATTGTCAATCTTCATTCAGCATTTCC	AbiEi-A3 R primer	MfeI
PF1607	TTTGAATTCAGGAGAAATTAACTATGGATA AGCATTCACCTTATTATC	AbiE-T2 F primer	EcoRI
PF1608	TTTTCTAGACTACTCTATCCACTCGGCAAG	AbiE-T2 R primer	XbaI
PF1609	TTTGAATTCAGGAGAAATTAACTATGCTGA AGCAACAAATTCG	AbiE-T1 F primer	EcoRI
PF1610	TTTTCTAGAGTTCGAATATTCAGAACTAGC CC	AbiE-T1 R primer	XbaI
PF1611	TTTGTCGACAGGAGAAATTAACTATGAAGA TTGACCCGGC	AbiE-T3 F primer	SalI
PF1612	TTTTCTAGATTAAAGTTTGAATTCAGGCTCA G	AbiE-T3 R primer	XbaI
PF1679	TTTGAATTCGAGCGTGTAGGAGATAAAGTA TGC	F AbiE-T1 Native RBS for pBAD30	EcoRI
PF1680	TTTGGATCCAAGGAAATGCTGAATGAAGAT TG	F AbiE-T3 with native RBS for pBAD30	BamHI
PF1677	TTTGAATTCCTATGAAGTTATAGACAGGTC AAATACTG	F 200bp upstream of AbiE-3	EcoRI
PF1678	TTTGGTACCTCCGCTCGGTAAGACGC	F 200bp upstream of AbiE-1	KpnI
PF3140	GTGACTGGAGTTCAGACGTGTGCTCTTCC GATC*T	Tnseq 1st round enrichment primer, binds NEB Illumina adapter sequence (no index or P7)	
PF3139	**/5BiotinTEG/TCATCTGCAG**CCGGGAATTCT CATGTTTGACA*G	Tnseq 1st round enrichment primer, binds Tn (biotinylated, no P5)	
PF3270	AATGATACGGCGACCACCGAGATCT**ACAC** CGCGCGATAAATCTAGAGTCGACCT*G	Tnseq second round nested enrichment primer	
PF3124	AATGATACGGCGACCACCGAG	P5 illumina site qPCR primer	
PF3125	CAAGCAGAAGACGGCATACGA	P7 illumina site qPCR primer	

Primer	Sequence (5'-3')	Notes[a]	Restriction Sites
PF2926	CAGGCATGCAAGCTTCAGGGTTGAGATGTG	Custom sequencing primer Tn-Seq (binds transposon)	
PF3441	ACACTCTTTCCCTACACGACGCTCTTCCGATCT	Illumina Read 1 primer	

[a] F denotes forward, R denotes reverse, LHF denotes left hand flank and RHF denotes right hand flank.

Table S4. Nucleotide differences between WT and LacA *Serratia* strains

**Nucleotide position**	**WT nt**	**LacA nt**	**Amino acid change**	**Gene**
950,750	G	T	Gly – Val	Enoyl-CoA hydratase/isomerase family protein
1,337,554	C	T	Pro – Leu	Cell division protein FtsQ
1,648,109	C	T	Ser – Leu	Allantoinase PuuE
1,696,583	G	A	Cys - Tyr	Ig domain protein group 1 domain protein
1,773,254	G	A	Gly – Glu	Phosphatidylserine decarboxylase family protein
2,074,568	G	T	Arg – Ile	HAAAP family serine/threonine permease
2,075,851	G	A	NA	Non-coding region
2,532,353	G	A	Gly -Glu	FliC/FljB family flegellin
3,816,858	A	- (deletion)	Stop	LacZ
4,683,680	C	T	Ser - Leu	Amino acid deaminase

Table S5. Genomic Islands predicted by Islandviewer in *Serratia*

Island number	Island start	Island end	Locus tags (CWC46_#)	Predicted Gene product
GI_1	234802	254403	01075	YicC family protein
			01080	site-specific integrase
			01085	hypothetical protein
			01090	**VapC-1**
			01095	**Irr like HTH domain**
			01100	AlpA family phage regulatory protein
			01105	Fic family protein
			01110	**HipA-2**
			01115	**HipB-2**
			01120	**AbiE-T1**
			01125	**AbiE-A1**
			01130	methyltransferase domain-containing protein
			01135	hypothetical protein
			01140	SEC-C motif domain protein
			01145	**HipA-3**
			01150	**HipB-3**
			01155	transcriptional regulator
			01160	PLP-dependent aminotransferase family protein
			01165	ABC-type transporter, periplasmic subunit
GI_2	787596	795797	03630	TonB-dependent vitamin B12 receptor BtuB
			03635	glutamate racemase
			03640	hypothetical protein
			03680	hypothetical protein
GI_3	846499	857764	03900	protein of unknown function DUF6 transmembrane
			03905	class I SAM-dependent methyltransferase
			03910	thymidylate synthase
			03915	nucleoside 2-deoxyribosyltransferase
			03920	NUDIX domain-containing protein
			03925	MFS transporter
			03930	gfo/Idh/MocA family oxidoreductase
			03935	acyltransferase
			03940	MerR family DNA-binding transcriptional regulator
			03945	alpha/beta hydrolase
			03950	3-dehydroquinate synthase
GI_4	951245	961936	04350	LysR family transcriptional regulator
			04355	glutathione S-transferase
			04360	alkyl hydroperoxide reductase
			04365	KR domain-containing protein
			04370	integrase
			04375	IS3 family transposase
			04380	integrase
			04385	hypothetical protein
			04390	hypothetical protein
			04395	hypothetical protein
			04400	hypothetical protein
			04405	hypothetical protein
			04410	**RelB/ParD-3**
			04415	**RelE/ParE-3**
			04420	hypothetical protein
GI_5	1402026	1407731	06430	glycosyl transferase family 2
			06435	polysaccharide biosynthesis protein
			06440	glycosyltransferase family 1 protein
			06445	glycosyl transferase group 1
			06450	amino acid ABC transporter permease
			06455	amino acid ABC transporter permease
GI_6 (SP1)	1688948	1736642	07760	hypothetical protein
			07765	class I SAM-dependent methyltransferase
			07770	hypothetical protein
			07775	hypothetical protein
			07780	recombination protein NinB
			07785	Lambda NinG family protein
			07790	antitermination Q family protein
			07795	site-specific DNA-methyltransferase
			07800	Lysis S family protein

Island number	Island start	Island end	Locus tags (CWC46_#)	Predicted Gene product
			07805	lysozyme
			07810	hypothetical protein
			07815	Ig domain protein group 1 domain protein
			07820	hypothetical protein
			07825	hypothetical protein
			07830	PBSX family phage terminase large subunit
			07835	DUF1073 domain-containing protein
			07840	phage Mu F like family protein
			07845	DUF2213 domain-containing protein
			07850	hypothetical protein
			07855	DUF2184 domain-containing protein
			07860	hypothetical protein
			07865	DUF4054 domain-containing protein
			07870	hypothetical protein
			07875	hypothetical protein
			07880	hypothetical protein
			07885	DUF3383 domain-containing protein
			07890	DUF3277 domain-containing protein
			07895	hypothetical protein
			07900	hypothetical protein
			07905	lytic transglycosylase catalytic
			07910	hypothetical protein
			07915	hypothetical protein
			07920	hypothetical protein
			07925	hypothetical protein
			07930	DUF2335 domain-containing protein
			07935	phage baseplate assembly protein V
			07940	hypothetical protein
			07945	hypothetical protein
			07950	DUF2612 domain-containing protein
			07955	hypothetical protein
			07960	tail fiber assembly protein
			07965	integrase
			07970	IS110 family transposase
			07975	IS3 family transposase
			07980	RNA-directed DNA polymerase
			07985	hypothetical protein
			07990	hypothetical protein
			07995	transcriptional regulator
			08000	arsenical resistance operon transcriptional repressor ArsD
			08005	arsenical pump-driving ATPase *arsA*
			08010	arsenical efflux pump membrane protein ArsB
			08015	arsenate reductase (glutaredoxin) *arsC*
			08020	hypothetical protein
			08025	phosphotransferase system EIIC
			08030	6-phospho-beta-glucosidase
			08035	LysR family transcriptional regulator
			08040	monosaccharide-transporting ATPase
			08045	Periplasmic binding protein domain containing protein
			08050	DUF6 domain-containing protein
			08055	DUF1203 domain-containing protein
GI_7	1916335	1931526	08930	hemagglutinin
GI_8	1990121	2020922	09195	hypothetical protein
			09200	aldehyde dehydrogenase (NAD(+))
			09205	IS3 family transposase
			09210	KR domain-containing protein
			09215	transcriptional regulator, BadM/Rrf2 family
			09220	IS256 family transposase
			09225	hypothetical protein
			09230	redox-sensitive transcriptional activator SoxR
			09235	major facilitator transporter
			09240	MFS transporter
			09245	IS3 family transposase ISHar7
			09250	AraC family transcriptional regulator
			09255	RadC-like JAB domain containing protein
			09260	DUF3363 domain-containing protein

Island number	Island start	Island end	Locus tags (CWC46_#)	Predicted Gene product
			09265	transcriptional regulator
			09270	LysR family transcriptional regulator
			09275	flavodoxin family protein
			09280	hypothetical protein
			09285	RimK family alpha-L-glutamate ligase
			09290	hypothetical protein
			09295	DegT/DnrJ/EryC1/StrS family aminotransferase
			09300	N-acetyl-gamma-glutamyl-phosphate reductase *argC*
			09305	N-acetyl-ornithine/N-acetyl-lysine deacetylase
			09310	transketolase
			09315	transketolase
			09320	MFS transporter
			09325	ATP-grasp domain-containing protein
			09330	IS3 family transposase
			09335	NAD(P)-dependent oxidoreductase
			09340	IS66 family insertion sequence hypothetical protein
			09345	IS66 family insertion sequence hypothetical protein
			09350	hypothetical protein
			09355	IS3 family transposase
			09360	LysR family transcriptional regulator
			09365	hypothetical protein
			09370	exonuclease ABC subunit UvrA
			09375	MarR family transcriptional regulator
			09380	ABC transporter substrate-binding protein
GI_9	2177006	2191834	10120	aminotransferase class V-fold PLP-dependent enzyme
			10125	hypothetical protein
			10130	AlpA family phage regulatory protein
			10135	DNA-directed RNA polymerase subunit beta
			10140	hypothetical protein
			10145	DUF4062 domain-containing protein
			10150	hypothetical protein
			10155	IS66 family insertion sequence hypothetical protein
			10160	IS66 family transposase ISPlu20
			10165	hypothetical protein
			10170	hypothetical protein
			10175	DUF4102 domain-containing protein
			10185	phospholipid-binding lipoprotein MlaA
GI_10	2180607	2188561	10140	hypothetical protein
			10145	DUF4062 domain-containing protein
			10150	hypothetical protein
			10155	IS66 family insertion sequence hypothetical protein
			10160	IS66 family transposase ISPlu20
			10165	hypothetical protein
			10170	hypothetical protein
GI_11	2373249	2381908	10885	PRD domain-containing protein
			10890	PTS beta-glucoside transporter subunit EIIBCA
			10895	6-phospho-beta-glucosidase
			10900	EamA/RhaT family transporter
			10905	hypothetical protein
			10910	N-acetyltransferase
			10915	DUF81 domain-containing protein
			10920	PLP-dependent aminotransferase family protein
GI_12	2537729	2548556	11580	hypothetical protein
			11585	DegT/DnrJ/EryC1/StrS family aminotransferase
			11590	class I SAM-dependent methyltransferase
			11595	ATP-grasp domain-containing protein
			11600	gfo/Idh/MocA family oxidoreductase
			11605	radical SAM protein
GI_13	2863918	2892043	12890	***symE*-1**
			12895	***symE*-1 XRE transcriptional regulator, HTH domain**
			12900	TOPRIM domain-containing protein
			12905	integrase
			12910	hypothetical protein
			12915	hypothetical protein
			12920	***symE*-2**
			12925	***symE*-2 XRE transcriptional regulator, HTH domain**

Island number	Island start	Island end	Locus tags (CWC46_#)	Predicted Gene product
			12930	TOPRIM domain-containing protein
			12935	integrase
			12940	hypothetical protein
			12945	hypothetical protein
			12950	hypothetical protein
			12955	***symE*-3**
			12960	hypothetical protein
			12965	hypothetical protein
			12970	hypothetical protein
			12975	hypothetical protein
			12980	hypothetical protein
			12985	***symE*-4**
			12990	***symE*-5**
			12995	hypothetical protein
			13000	**ParD-1**
			13005	**ParE-1**
			13010	***symE*-6**
			13015	recombinase XerD
			13020	ASCH domain-containing protein
			13025	FAD-binding oxidoreductase
			13030	ABC transporter substrate-binding protein
			13035	hypothetical protein
			13040	iron ABC transporter permease
			13045	YrhK domain containing protein
			13050	GGDEF domain-containing protein
			13055	glycine dehydrogenase
			13060	hypothetical protein
GI_14 (SP2)	3142496	3165816	14140	hypothetical protein
			14145	hypothetical protein
			14150	hypothetical protein
			14155	glycoside hydrolase family 24
			14160	hypothetical protein
			14165	phage anti-repressor protein
			14170	Anti-repressor
			14175	Arc family DNA-binding protein
			14180	Arc family DNA-binding protein
			14185	hypothetical protein
			14190	hypothetical protein
			14195	DUF3383 domain-containing protein
			14200	hypothetical protein
			14205	hypothetical protein
			14210	hypothetical protein
			14215	DUF4054 domain-containing protein
			14220	hypothetical protein
			14225	DUF2184 domain-containing protein
			14230	hypothetical protein
			14235	DUF2213 domain-containing protein
			14240	phage head morphogenesis protein, SPP1 gp7 family
			14245	DUF1073 domain-containing protein
			14250	hypothetical protein
			14255	terminase small subunit
			14260	hypothetical protein
			14265	hypothetical protein
			14270	hypothetical protein
			14275	hypothetical protein
			14280	hypothetical protein
			14285	peptidase
			14290	hypothetical protein
			14295	lysozyme
			14300	Lysis S family protein
			14305	**HicB-2**
			14310	**HicA-2**
GI_15	3307897	3312428	15075	DUF4440 domain-containing protein
			15080	hypothetical protein
			15085	hypothetical protein
			15090	hypothetical protein

Island number	Island start	Island end	Locus tags (CWC46_#)	Predicted Gene product
			15095	hypothetical protein
			15100	hypothetical protein
			15105	**HigB-1**
			15110	**HigA-1**
			15115	integrase
GI_16	3652781	3685478	16695	gluconeogenesis factor
			16700	exonuclease ABC subunit B
			16705	molybdenum-pterin-binding protein
			16710	IS3 family transposase
			16715	AAA ATPase
			16720	DUF4435 domain-containing protein
			16725	IS4 family transposase
			16730	IS3 family transposase
			16735	IS3 family transposase
			16740	SEC-C motif domain protein
			16745	hypothetical protein
			16750	DNA primase
			16755	hypothetical protein
			16760	IS3 family transposase
			16780	CDP-diacylglycerol--glycerol-3-phosphate 3-phosphatidyltransferase
			16785	exonuclease ABC subunit UvrC
			16790	two-component system response regulator UvrY
			16800	D-serine/D-alanine/glycine transporter
			16805	LysR family transcriptional regulator
			16815	integrase
			16820	glutathione peroxidase
			16825	NADP-dependent oxidoreductase
			16830	LysR family transcriptional regulator
			16835	6-phospho-beta-glucosidase
			16840	PTS lactose/cellobiose transporter subunit IIA
			16845	PTS sugar transporter subunit IIC
			16850	PTS sugar transporter subunit IIB
			16855	GntR family transcriptional regulator
			16860	IS30 family transposase
			16865	alcohol dehydrogenase
GI_17	3788255	3793938	17310	hydrogenase maturation peptidase HycI
			17315	cysteine synthase family protein
			17320	diaminopimelate decarboxylase *lysA*
			17325	DUF340 domain-containing protein
			17330	alanyl-tRNA editing protein
			17335	NAD(P)-dependent oxidoreductase
			17340	aminoglycoside phosphotransferase
GI_18	4348689	4431319	19760	tRNA-Phe
			19765	site-specific integrase
			19770	PFAM07515 Putative conjugal transfer nickase/helicase TraI C-term
			19775	Hypothetical protein
			19780	Hypothetical protein
			19785	Conjugal transfer protein TraF
			19790	Hypothetical protein
			19795	Hypothetical protein
			19800	SAM-dependent DNA methyltransferase
			19805	Hypothetical protein
			19810	DUF1281 domain-containing protein
			19815	**CbtA -1**
			19820	**YeeU-1**
			19825	hypothetical protein
			19830	hypothetical protein
			19835	IS3 family transposase
			19840	LysE family translocator
			19845	hypothetical protein
			19850	integrase catalytic region
			19855	hypothetical protein
			19860	integrase catalytic domain
			19865	hypothetical protein

Island number	Island start	Island end	Locus tags (CWC46_#)	Predicted Gene product
			19870	hypothetical protein
			19875	hypothetical protein
			19880	hypothetical protein
			19885	hypothetical protein
			19890	*cas1*
			19895	*cas2*
			19900	*cas10*
			19905	*csm2*
			19910	*csm3*
			19915	*csm4*
			19920	*csm5*
			19925	*cas6*
			19930	*nucC*-like accessory nuclease
			19935	hypothetical protein
			19940	conjugal transfer protein TraG
			19945	hypothetical protein
			19950	integrating conjugative element protein
			19955	TIGR03756 family integrating conjugative element protein
			19960	TIGR03757 family integrating conjugative element protein
			19965	**RelB/ParD-4**
			19970	**RelE/ParE-4**
			19975	Hypothetical protein
			19980	Conjugative transfer ATPase
			19985	TIGR03751 family conjugal transfer lipoprotein
			19990	TIGR03752 family integrating conjugative element protein
			19995	TIGR03749 family integrating conjugative element protein
			20000	TIGR03746 family integrating conjugative element protein
			20005	TIGR03750 family conjugal transfer protein
			20010	TIGR03745 family integrating conjugative element membrane protein
			20015	TIGR03758 family integrating conjugative element protein
			20020	Protein of unknown function
			20025	Hypothetical protein
			20030	PerC family transcriptional regulator
			20035	TIGR03747 family integrating conjugative element membrane protein
			20040	*traD*
			20045	restriction endonuclease
			20050	Integrating conjugative element protein
			20055	Lytic transglycosylase domain containing protein
			20060	TIGR03759 family integrating conjugative element protein
			20065	Hypothetical protein
			20070	shufflon system plasmid conjugative transfer pilus tip adhesin PilV
			20075	prepilin peptidase
			20080	Lytic transglycosylase catalytic
			20085	PilS domain-containing protein
			20090	pilus assembly protein PilR
			20095	type II secretion system protein E
			20100	*pilP*
			20105	20105
			20110	PilN family type IVB pilus formation outer membrane protein
			20115	PilM inner membrane family protein
			20120	Integrating conjugative element protein PilL, PFGI-1
			20125	DUF29 domain-containing protein
			20130	single-stranded DNA-binding protein
			20135	DUF3577 domain-containing protein
			20140	DNA topoisomerase III
			20145	TIGR03761 family integrating conjugative element protein
			20150	Hypothetical protein
			20155	Hypothetical protein
			20160	Hypothetical protein
			20165	DUF2857 domain-containing protein
			20170	DUF2786 domain-containing protein
			20175	conjugal transfer protein TraR
			20180	chromosome partitioning protein ParB

Island number	Island start	Island end	Locus tags (CWC46_#)	Predicted Gene product
			20185	*dnaB*
			20190	ParA family protein
GI_19	4360699	4367681	19830	hypothetical protein
			19835	IS3 family transposase
			19840	LysE family translocator
			19845	hypothetical protein
GI_20	4623354	4636001	21085	DUF4102 domain-containing protein
			21090	hypothetical protein
			21095	RNA-directed DNA polymerase
			21100	prepilin cleavage protein
			21105	hypothetical protein
			21110	hypothetical protein
			21115	hypothetical protein
			21120	MobA/MobL protein
			21125	hypothetical protein
			21130	hypothetical protein
			21135	hypothetical protein
GI_21	4720040	4752046	21540	glycosyltransferase family 1 protein
			21545	NAD-dependent epimerase
			21550	GDP-mannose 4,6-dehydratase *gmd*
			21555	hypothetical protein
			21560	hypothetical protein
			21565	transposase
			21570	glycosyl transferase group 1
			21575	glycosyl transferase family 2
			21580	methyltransferase domain-containing protein
			21585	IS66 family transposase ISPlu20
			21590	IS66 family insertion sequence hypothetical protein
			21595	hypothetical protein
			21600	hypothetical protein
			21605	FAD-dependent oxidoreductase
			21610	UPF0104 family protein
			21615	glucose-1-phosphate cytidylyltransferase *rfbF*
			21620	glycosyltransferase
			21625	NAD(P)-dependent oxidoreductase
			21630	CDP-glucose 4,6-dehydratase *rbfG*
			21635	hypothetical protein
			21640	hypothetical protein
			21645	class I SAM-dependent methyltransferase
			21650	ABC transporter ATP-binding protein
			21655	ABC transporter permease
			21660	phosphomannomutase/phosphoglucomutase
			21665	mannose-1-phosphate guanylyltransferase/mannose-6-phosphate isomerase
			21670	dTDP-4-dehydrorhamnose 3,5-epimerase *rfbC*
GI_22	4733518	4747692	21590	IS66 family insertion sequence hypothetical protein
			21595	hypothetical protein
			21600	hypothetical protein
			21605	FAD-dependent oxidoreductase
			21610	Lysylphosphatidyl glycerol synthetase family protein
			21615	glucose-1-phosphate cytidylyltransferase *rfbF*
			21620	glycosyltransferase
			21625	NAD(P)-dependent oxidoreductase
			21630	CDP-glucose 4,6-dehydratase *rfbG*
			21635	hypothetical protein
			21640	hypothetical protein
			21645	class I SAM-dependent methyltransferase
			21650	ABC transporter ATP-binding protein
			21655	ABC transporter permease

Table S6. ***Serratia*** **contains three prophages predicted by PHASTER**

Old prophage Name	New Name	Length (Kb)	Total Proteins	Phage hit proteins	Hypothetical proteins	Bacterial proteins	*att* site	GC%
PHAGE-Salmon-SEN34_NC_028699(23)	SP1	42.5	59	53	2	4	yes	51
PHAGE_Pectob_ZF40_NC_019522(32)	SP2	49.3	70	55	6	9	yes	49
PHAGE_Entero_PsP3_NC_005340(11)	SP3	37.3	42	42	35	2	no	48

Table S7. Differentially expressed genes in response to AbiE-T2 at 5 min post toxin induction

Locus tag	Gene name	Base mean	Log2fold change	padj	Notes
CWC46_RS04260	AbiE-T2	14195.92	7.42	8.74×10^{-269}	
CWC46_RS04265	AbiE-A2	764.53	3.93	6.16×10^{-59}	
CWC46_RS22245	pectate lyase	276.36	0.80	3.81×10^{-4}	
CWC46_RS13985	tRNA-Tyr	609.51	0.67	1.76×10^{-3}	
CWC46_RS09915	*carA*	18280.07	-1.10	3.71×10^{-3}	
CWC46_RS18020	tRNA-Lys	320.18	0.84	3.71×10^{-3}	
CWC46_RS20680	*pigE*	747.93	-0.60	5.12×10^{3}	
CWC46_RS09920	*carB*	5965.64	-1.01	9.81×10^{-3}	
CWC46_RS08800	tRNA-Val	1877.99	0.74	1.38×10^{-2}	
CWC46_RS22415	tRNA-Phe	666.79	0.80	1.37×10^{-2}	
CWC46_RS08810	tRNA-Lys	1117.26	0.61	1.42×10^{-2}	
CWC46_RS12210	tRNA-Val	378.35	0.95	1.74×10^{-2}	
CWC46_RS20685	*pigD*	520.62	-0.73	2.26×10^{-2}	
CWC46_RS09935	*carE*	1315.42	-0.74	2.32×10^{-2}	
CWC46_RS07870	Hypothetical protein	56.26	1.07	2.39×10^{-2}	SP1
CWC46_RS18015	tRNA-Lys	432.49	0.69	2.55×10^{-2}	
CWC46_RS08525	tRNA-Asp	782.97	0.74	2.91×10^{-2}	
CWC46_RS00145	tRNA-Leu	533.44	0.73	3.11×10^{-2}	
CWC46_RS03865	tRNA-His	2136.48	0.66	3.11×10^{-2}	
CWC46_RS22315	Hypothetical protein	1246.45	-0.83	3.26×10^{-2}	DUF1469
CWC46_RS14335	Hypothetical protein	297.57	0.67	3.60×10^{-2}	SP2
CWC46_RS07780	recombination protein NinB	41.93	1.09	4.45×10^{-2}	SP1
CWC46_RS18435	Hypothetical protein	111.97	0.92	5.65×10^{-2}	
CWC46_RS20785	Taurine catabolism dioxygenase TauD/TfdA CDS	267.29	-0.68	5.65×10^{-2}	
CWC46_RS06385	sn-glycerol-3-phosphate ABC transporter ATP-binding protein UgpC CDS	211.35	-0.94	5.73×10^{-2}	
CWC46_RS07775	hypothetical protein	217.13	0.60	5.73×10^{-2}	SP1
CWC46_RS07860	hypothetical protein	17.02	1.66	5.73×10^{-2}	
CWC46_RS08805	tRNA-Val	2242.73	0.67	5.73×10^{-2}	
CWC46_RS09930	*carD*	3184.39	-0.85	5.73×10^{-2}	
CWC46_RS11620	phosphate starvation-inducible protein PhoH	1215.64	-0.5	5.73×10^{-2}	SP1
CWC46_RS14975	transcriptional activator Ogr/delta	347.51	0.57	5.73×10^{-2}	SP3
CWC46_RS15025	MFS transporter	165.38	-0.65	5.73×10^{-2}	
CWC46_RS16770	tRNA-Cys	2335.00	0.66	5.73×10^{-2}	
CWC46_RS16795	tRNA-Lys	93.84	0.75	5.73×10^{-2}	
CWC46_RS21830	LysR family transcriptional regulator	310.32	-0.59	5.73×10^{-2}	
argH	argH	30.87	1.26	5.73×10^{-2}	

Locus tag	Gene name	Base mean	Log2fold change	padj	Notes
CWC46_RS14330	DUF1367 domain-containing protein	358.36	0.6	7.08×10^{-2}	SP2
CWC46_RS20675	*pigF*	295.94	-0.5	7.10×10^{-2}	
CWC46_RS07740	DUF1019	300.67	0.77	7.23×10^{-2}	
CWC46_RS03860	tRNA-Arg	1647.35	0.65	7.61×10^{-2}	
CWC46_RS03875	tRNA-Pro	1446.61	0.62	7.61×10^{-2}	
CWC46_RS07805	lysozyme	63.29	1.12	7.61×10^{-2}	SP1
CWC46_RS07810	hypothetical protein	86.02	1.11	7.61×10^{-2}	SP1
CWC46_RS07885	DUF3383	60.20	1.03	7.61×10^{-2}	SP2
CWC46_RS08320	tRNA-Arg	2306.92	0.53	7.61×10^{-2}	
CWC46_RS09925	*carC*	4754.71	-0.86	7.61×10^{-2}	
CWC46_RS09940	*carF*	2169.70	-0.82	7.61×10^{-2}	
CWC46_RS21820	acetolactate synthase AlsS	495.09	-0.80	7.61×10^{-2}	
CWC46_RS22205	30S ribosomal protein S21	17176.45	0.69	7.61×10^{-2}	
CWC46_RS07645	DNA polymerase IV	406.62	0.47	7.73×10^{-2}	
CWC46_RS08325	tRNA-Arg	2781.71	0.42	7.773×10^{-2}	
CWC46_RS18010	RtT sRNA	556.07	0.49	7.73×10^{-2}	
CWC46_RS19760	tRNA-Phe	1777.09	0.75	8.18×10^{-2}	
CWC46_RS09950	*carH*	1160.42	-0.80	8.44×10^{-2}	
CWC46_RS02245	tRNA-Glu	4563.74	0.50	8.50×10^{-2}	
CWC46_RS02580	Pseudogene	88.68	0.69	8.50×10^{-2}	
CWC46_RS02615	acyl carrier protein CDS	777.65	-0.46	8.50×10^{-2}	
CWC46_RS07835	DUF1073	148.41	0.85	8.50×10^{-2}	SP1
CWC46_RS08330	tRNA-Arg	668.07	0.47	8.50×10^{-2}	
CWC46_RS15055	cupin domain-containing protein	165.68	-0.68	8.50×10^{-2}	
gspJ	gspJ	117.92	-0.66	8.50×10^{-2}	
CWC46_RS05435	amidohydrolase	52.97	-1.32	8.73×10^{-2}	
CWC46_RS07735	XRE family transcriptional regulator	173.76	0.78	8.79×10^{-2}	SP1
CWC46_RS15060	TetR/AcrR family transcriptional regulator	179.94	-0.64	8.83×10^{-2}	
CWC46_RS02440	tRNA-Glu	4515.0	0.49	9.59×10^{-2}	
CWC46_RS01405	MltR family transcriptional repressor	430.90	0.59	9.87×10^{-2}	

Table S8. Differentially expressed genes in response to AbiE-T2 at 20min post toxin induction

Locus tag	Gene name	Base mean	Log2fold change	padj	Notes
CWC46_RS04260	AbiE-T2	102559.34	10.01	0	
CWC46_RS04265	AbiE-A2	3202.07	5.71	2.9×10^{-61}	
CWC46_RS07750	hypothetical protein	190.14	1.15	2.52×10^{-3}	SP1
CWC46_RS07835	DUF1073 domain containing protein	181.67	1.72	2.52×10^{-3}	SP1
CWC46_RS14945	phage major tail tube protein	2443.99	0.70	3.87×10^{-2}	SP3
CWC46_RS09930	*carD*	1356.10	-1.52	4.83×10^{-2}	
CWC46_RS09920	*carB*	3084.34	-1.58	6.77×10^{-2}	
CWC46_RS07810	hypothetical protein	88.66	1.65	8.03×10^{-2}	SP1
CWC46_RS07830	PBSX family phage terminase	112.45	1.39	8.03×10^{-2}	SP1
CWC46_RS07885	DUF3383 domain containing protein	78.13	1.43	8.03×10^{-2}	SP1
CWC46_RS07945	Hypothetical protein	105.34	2.03	8.03×10^{-2}	SP1
CWC46_RS09915	*carA*	6956.72	-1.39	8.03×10^{-2}	
CWC46_RS09925	*carC*	3688.35	-1.66	8.03×10^{-2}	
ffs	*ffs* ncRNA	1447.48	1.65	8.03×10^{-2}	
CWC46_RS07740	protein of unknown function DUF1019	264.89	0.91	9.99×10^{-2}	SP1
CWC46_RS07845	DUF2213 domain containing protein	100.30	1.33	9.99×10^{-2}	
CWC46_RS09935	*carE*	694.26	-1.17	9.99×10^{-2}	

Supplementary References

1. Garcia-Pino, A., Christensen-Dalsgaard, M., Wyns, L., Yarmolinsky, M., Magnuson, R.D., Gerdes, K. and Loris, R. (2008) Doc of prophage P1 is inhibited by its antitoxin partner Phd through fold complementation. *J Biol Chem*, **283**, 30821-30827.
2. Masuda, H., Tan, Q., Awano, N., Wu, K.P. and Inouye, M. (2012) YeeU enhances the bundling of cytoskeletal polymers of MreB and FtsZ, antagonizing the CbtA (YeeV) toxicity in *Escherichia coli*. *Mol Microbiol*, **84**, 979-989.
3. Masuda, H., Tan, Q., Awano, N., Yamaguchi, Y. and Inouye, M. (2012) A novel membrane-bound toxin for cell division, CptA (YgfX), inhibits polymerization of cytoskeleton proteins, FtsZ and MreB, in *Escherichia coli*. *FEMS Microbiol Lett*, **328**, 174-181.
4. McNeil, M.B., Iglesias-Cans, M.C., Clulow, J.S. and Fineran, P.C. (2013) YgfX (CptA) is a multimeric membrane protein that interacts with the succinate dehydrogenase assembly factor SdhE (YgfY). *Microbiology*, **159**, 1352-1365.
5. Wang, X., Lord, D.M., Cheng, H.Y., Osbourne, D.O., Hong, S.H., Sanchez-Torres, V., Quiroga, C., Zheng, K., Herrmann, T., Peti, W. *et al.* (2012) A new type V toxin-antitoxin system where mRNA for toxin GhoT is cleaved by antitoxin GhoS. *Nat Chem Biol*, **8**, 855-861.
6. Marimon, O., Teixeira, J.M., Cordeiro, T.N., Soo, V.W., Wood, T.L., Mayzel, M., Amata, I., Garcia, J., Morera, A., Gay, M. *et al.* (2016) An oxygen-sensitive toxin-antitoxin system. *Nat Commun*, **7**, 13634.
7. Li, H. (2018) Minimap2: pairwise alignment for nucleotide sequences. *Bioinformatics*, **34**, 3094-3100.
8. Li, H., Handsaker, B., Wysoker, A., Fennell, T., Ruan, J., Homer, N., Marth, G., Abecasis, G., Durbin, R. and Genome Project Data Processing, S. (2009) The Sequence Alignment/Map format and SAMtools. *Bioinformatics*, **25**, 2078-2079.
9. Quinlan, A.R. and Hall, I.M. (2010) BEDTools: a flexible suite of utilities for comparing genomic features. *Bioinformatics*, **26**, 841-842.
10. R Core team. (2019) R: A language and environment for statistical computaing.
11. Will, S., Joshi, T., Hofacker, I.L., Stadler, P.F. and Backofen, R. (2012) LocARNA-P: accurate boundary prediction and improved detection of structural RNAs. *RNA*, **18**, 900-914.
12. Raden, M., Ali, S.M., Alkhnbashi, O.S., Busch, A., Costa, F., Davis, J.A., Eggenhofer, F., Gelhausen, R., Georg, J., Heyne, S. *et al.* (2018) Freiburg RNA tools: a central online resource for RNA-focused research and teaching. *Nucleic Acids Res*, **46**, W25-W29.
13. Thoma, S. and Schobert, M. (2009) An improved *Escherichia coli* donor strain for diparental mating. *FEMS Microbiol Lett*, **294**, 127-132.
14. Thomson, N.R., Crow, M.A., McGowan, S.J., Cox, A. and Salmond, G.P.C. (2000) Biosynthesis of carbapenem antibiotic and prodigiosin pigment in *Serratia* is under quorum sensing control. *Molecular Microbiology*, **36**, 539-556.
15. Richter, C., Dy, R.L., McKenzie, R.E., Watson, B.N., Taylor, C., Chang, J.T., McNeil, M.B., Staals, R.H. and Fineran, P.C. (2014) Priming in the Type I-F

CRISPR-Cas system triggers strand-independent spacer acquisition, bi-directionally from the primed protospacer. *Nucleic Acids Res*, **42**, 8516-8526.
16. Yanisch-Perron, C., Viera, J. and Messing, J. (1985) Improved M13 phage cloning vectors and host strains: nuelcotide sequences of the M13mp18 and pUC19 vectors. *Gene*, 103-119.
17. Guzman, L.M., Belin, D., Carson, M.J. and Beckwith, J. (1995) Tight regulation, modulation, and high-level expression by vectors containing the arabinose PBAD promoter. *J Bacteriol*, **177**, 4121-4130.
18. Dy, R.L., Przybilski, R., Semeijn, K., Salmond, G.P. and Fineran, P.C. (2014) A widespread bacteriophage abortive infection system functions through a Type IV toxin-antitoxin mechanism. *Nucleic Acids Res*, **42**, 4590-4605.
19. Patterson, A.G., Chang, J.T., Taylor, C. and Fineran, P.C. (2015) Regulation of the Type I-F CRISPR-Cas system by CRP-cAMP and GalM controls spacer acquisition and interference. *Nucleic Acids Res*, **43**, 6038-6048.
20. Kitagawa, M., Ara, T., Arifuzzaman, M., Ioka-Nakamichi, T., Inamoto, E., Toyonaga, H. and Mori, H. (2005) Complete set of ORF clones of *Escherichia coli* ASKA library (a complete set of E. coli K-12 ORF archive): unique resources for biological research. *DNA Res*, **12**, 291-299.
